# Copper-mediated thiol potentiation and mutagenesis-guided modeling suggest a highly conserved copper-binding motif in human OR2M3

**DOI:** 10.1007/s00018-019-03279-y

**Published:** 2019-08-21

**Authors:** Franziska Haag, Lucky Ahmed, Krystle Reiss, Eric Block, Victor S. Batista, Dietmar Krautwurst

**Affiliations:** 1 Leibniz-Institute for Food Systems Biology, Technical University of Munich, Lise-Meitner-Str. 34, 85354 Freising, Germany; 2 Department of Chemistry, Yale University, New Haven, CT 06520 USA; 3 Department of Chemistry, University at Albany, State University of New York, Albany, NY 12222 USA

**Keywords:** GPCR, Structure–function study, Molecular modeling, Copper-binding motif, Silver ions

## Abstract

Sulfur-containing compounds within a physiological relevant, natural odor space, such as the key food odorants, typically constitute the group of volatiles with the lowest odor thresholds. The observation that certain metals, such as copper, potentiate the smell of sulfur-containing, metal-coordinating odorants led to the hypothesis that their cognate receptors are metalloproteins. However, experimental evidence is sparse—so far, only one human odorant receptor, OR2T11, and a few mouse receptors, have been reported to be activated by sulfur-containing odorants in a copper-dependent way, while the activation of other receptors by sulfur-containing odorants did not depend on the presence of metals. Here we identified an evolutionary conserved putative copper interaction motif CC/CSSH, comprising two copper-binding sites in TMH5 and TMH6, together with the binding pocket for 3-mercapto-2-methylpentan-1-ol in the narrowly tuned human receptor OR2M3. To characterize the copper-binding motif, we combined homology modeling, docking studies, site-directed mutagenesis, and functional expression of recombinant ORs in a cell-based, real-time luminescence assay. Ligand activation of OR2M3 was potentiated in the presence of copper. This effect of copper was mimicked by ionic and colloidal silver. In two broadly tuned receptors, OR1A1 and OR2W1, which did not reveal a putative copper interaction motif, activation by their most potent, sulfur-containing key food odorants did not depend on the presence of copper. Our results suggest a highly conserved putative copper-binding motif to be necessary for a copper-modulated and thiol-specific function of members from three subfamilies of family 2 ORs.

**Electronic supplementary material:**

The online version of this article (10.1007/s00018-019-03279-y) contains supplementary material, which is available to authorized users.

## Introduction

The perception of odors of all kinds is initiated by binding to G protein-coupled receptors (GPCRs), encoded by about 400 odorant receptor (OR) genes [1–3], of which at least 270 appear to be expressed in the cilia of olfactory sensory neurons (OSNs) within the main olfactory epithelium (OE) of the nasal cavity [4]. Odor coding is then achieved in a combinatorial manner whereby more ORs are broadly tuned than narrowly tuned and whereby one odorant may activate multiple ORs [5–14].

Thiols play an outstanding role in human olfaction, for instance as body odor [15–19], environmental odors [20], and especially as key food odorants (KFOs), which appear in foods at concentrations above their odor threshold, and critically determine the aroma of foods [21]. Thiol odorants often show very low odor thresholds [6, 21–25]. The olfactory sensitivity for thiols is shown not only for humans but also for spider monkeys (*Ateles geoffroyi*), squirrel monkeys (*Saimiri sciureus*), and pigtail macaque (*Macaca nemestrina*) [26]. Interestingly, these low odor thresholds have been associated with the presence of ions from transition metals such as copper, iron, zinc, or nickel [27–32]. Because of the remarkable olfactory potency of thiols, several theories have been put forward to explain this behavior.

In 1978, Crabtree [27] postulated that Cu(I) ions, because of their high affinity for thiols, coordinate them within the active center of odorant receptors, thereby constituting a sensitive thiol detector [27]. In the same year, Day [33] suggested that transition metals may be involved in the olfaction of certain functional groups, such as pyridines [33].

In 1996, Turin [34] published the so-called vibrational theory of olfaction [34], proposing that electron transfer, which is ubiquitous in biology, e.g., for photosynthesis, respiration, and nitrogen fixation, takes place in the OR protein by reducing the disulfide bond via a zinc ion [34].

In 2003, Wang et al. [28] postulated the “HxxC[DE]”-amino acid-motif (with x as a hydrophobic residue) in the second extracellular loop (ECL 2) of ORs to be crucially involved in the coordination of Cu^2+^- or Zn^2+^-ions and odorants within their receptors. They could observe a conformational change of this motif from pleated sheet to an α-helix in the presence of Zn^2+^, suggesting that ECL 2 becomes engaged in odorant binding [28]. The consensus sequence “HxxC[DE]” can be found in 74% of all human ORs, which led them to propose ORs as metalloproteins [28]. The role of metals in mammalian olfaction is the subject of recent reviews [35, 36].

In addition to ORs, other GPCRs have also been suggested to coordinate metal ions. For example, the binding of ligands in the opioid receptor is enhanced by manganese [37]. By introducing Cu^2+^, Zn^2+^, or Ni^2+^ ions into cyclam rings of AMD3100, the response of the CXCR4 chemokine receptor could be increased up to 50-fold [38]. Mutational analysis revealed that the enhancing effect could be eliminated by changing the single amino acid Asp262 in TMH 6 [38]. Furthermore, the two melanocortin receptors MC1 and MC4 have been shown to be enhanced by Zn^2+^ [39]. MC1 is expressed in melanocytes and controls skin tanning. MC4 expresses in certain regions of the hypothalamus in the brain, and within the intestinal tissue. It is involved in the regulation of autonomic responses as well as the regulation of energy homeostasis. Possible interaction sites were indicated as Cys271 (ECL 3) and Asp119 (extracellular end of TMH 3) [39]. Transition metals such as copper, zinc, and iron play an important role for the homeostasis of brain neurons [40, 41]. Aron et al. [42] showed that metals like copper can serve as dynamic signals that bind and regulate protein function at external allosteric sites in addition to their function as static metabolic cofactors [42].

Yokoi et al. [43] investigated dietary nickel deprivation on olfaction in rats and observed a decreased sniffing rate [43]. Since olfactory CNG channels are suppressed by nickel [44, 45], they suggested that nickel ions play a physiological role in olfactory function.

Viswaprakash et al. [46] reported zinc to enhance the odorant-induced responses in olfactory receptor neurons [46]. They observed an enhancement of signaling in these neurons, however, only using nanoparticles, but not using Zn^2+^ ions [46]. Furthermore, the use of copper, gold, or silver nanoparticles did not show a similar effect as compared to zinc nanoparticles [46]. Also Vodyanoy [47] investigated zinc nanoparticles, and came up with a model that predicted that one metal nanoparticle binds two receptor molecules to create a receptor dimer, which is consistent with the evidence that many GPCRs form dimers or larger oligomers [47]. In a later study, they showed that nanomolar suspensions of zinc nanoparticles enhance responses by a factor of 5 [48].

In 2012, Duan et al. [29] suggested Cu^2+^ ions to be an essential co-factor for the interaction of mouse OR Olfr1509 (MOR244-3) with its agonist (methylthio)methanethiol [29]. Since increasing the copper concentration in the cell-based assay led to a significant increase of the sensitivity of the receptor, whereas chelating agents decreased the receptor’s sensitivity, they postulated that thiols and copper ions form a complex, which renders the receptor very sensitive for thiols [29]. Based on this study, and by combining receptor modeling/ligand docking, site-directed mutagenesis, and functional expression of recombinant mutant OR, Sekharan et al. [30] identified three amino acid positions within TMH 3 and TMH 5, His105^3.33^, Cys109^3.37^, and Asn202^5.42^, which supposedly form a Cu-binding site within receptor Olfr1509 [30].

Most recently, Zhang et al. [32] de-orphanized another mouse OR for (methylthio)methanethiol which also shows a copper effect. For Olfr1019 (MOR180-1), the amino acid positions within TMH 5 and TMH 6, Cys203^5.42^, Met256^6.48^, and Arg261^6.53^, supposedly form a Cu-binding site within the receptor.

Furthermore, Li et al. [31] demonstrated that activation of human OR2T11 by small thiols mainly containing up to five carbon atoms depended on the presence of transition metal ions [31]. They identified two Cu-binding sites within OR2T11, which involve Met115^3.46^ of TMH 3, and residues Cys238^6.33^ and His241^6.35^ from TMH 6, or Met56^2.39^ of TMH 2, and Met133^4.37^, Arg135^4.39^, and Cys138^4.42^ of TMH 4 [31].

However, the mechanisms underlying the very sensitive detection of thiols by humans in general are still unsolved. So far, the Cu dependence of ORs’ responsiveness to thiols has been demonstrated for one human OR [31] and a few mouse ORs [29, 32]. Most of the thiol-responsive human ORs identified, so far are members of family 2 of ORs [5, 6, 11–13, 31, 49]. Recently, we identified one narrowly tuned thiol-responding human receptor, OR2M3, as well as two broadly tuned receptors with overlapping thiol agonist spectra [5, 6]. One causative mechanism for differences in tuning breadth may be the size of the respective ligand-binding pockets. Baud et al. suggested this for mouse receptors Olfr73 and Olfr74 [50]. Here, the ligand cavity size showed an accessible volume of 200 Å^3^ for broadly tuned Olfr73, and 250 Å^3^ for narrowly tuned Olfr74 [50].

We, therefore, hypothesized that rather narrowly tuned, thiol-specific ORs may exhibit a Cu potentiating effect on their responsiveness to thiols, and that these ORs have rather size-restricted binding pockets with limited degrees of freedom for alternative docking of thiols into their receptors. In contrast, broadly tuned ORs with larger binding pockets will lack a Cu potentiating effect on thiol activation, but, among many chemically diverse odorant agonists, may nevertheless also detect certain thiol structures.

Here, we investigated narrowly tuned OR2M3 with its agonist 3-mercapto-2-methylpentan-1-ol [6], a common and potent KFO from heated onions, which for thousands of years have been used worldwide as a food and in complementary medicine [51]. We compared OR2M3 with two most recently characterized broadly tuned receptors, OR2W1 and OR1A1, with three of their known agonists, 2-phenylethanethiol, 3-mercaptohexyl acetate, and allyl phenyl acetate [5], in a cell-based, online cAMP-luminescence GloSensor™ assay [52].

We used site-directed mutagenesis and functional expression of recombinant mutant ORs to investigate cognate human OR/KFO pairs in the presence and absence of Cu^2+^-ions. We rationalized the docking of specific thiols into the binding pockets of their respective ORs with QM/MM models involving chelation of copper by these thiols and compared the size of thiol-binding pockets between narrowly tuned thiol-specific OR2M3 and broadly tuned OR2W1. We found the effect of copper to be mimicked by ionic and colloidal silver.

## Materials and methods

### Chemicals

The following chemicals were used: Dulbecco´s MEM medium (#F0435), FBS superior (#S0615), L-glutamin (#K0282), penicillin (10 000U/mL)/streptomycin (10 000U/mL) (#A2212), trypsin/EDTA solution (#L2143) (Biochrom, Berlin, Germany), MEM non-essential amino acid solution (100x) (#M7145, Sigma-Aldrich, Steinheim, Germany), Gibco^®^ HAT supplement (#21060-017, Thermo Fisher, Dreieich, Germany), calcium chloride dihydrate (#22322.295), d-glucose (#101174Y), dimethyl sulfoxide (DMSO) (#83673.230), HEPES (#441476L), potassium chloride (#26764.230), and sodium hydroxide (#28244.295) (VWR Chemicals BDH Prolabo, Leuven, Belgium), sodium chloride (#1064041000, Merck, Darmstadt, Germany), D-luciferin (beetle) monosodium salt (#E464X, Promega, Madison, USA), copper(II) chloride (#751944), nickel(II) chloride hexahydrate (#31462), zinc sulfate heptahydrate (#Z0251), cobalt(II) chloride hexahydrate (#C8661), iron(III) chloride (#12321), colloidal silver (#85131), silver acetate (#8.01504.0005), silver nitrate (#1.01512.0025), tertaethylenepentamine pentahydrochloride (TEPA) (#375683, Sigma-Aldrich, Steinheim, Germany).

The odorants used were 3-mercapto-2-methylpentan-1-ol (#242803), 3-mercapothexyl acetate (#137912, Chemos GmbH, Regenstauf, Germany), 2-phenylethanethiol (#P1715, TCI Deutschland GmbH, Eschborn, Germany), allyl phenyl acetate (#W203904), and (*R*)-(-)-carvone (#124931, Sigma-Aldrich, Steinheim, Germany). All odorants were tested to be GC-O pure.

### Molecular cloning of human OR2M3, OR2W1, and OR1A1

The protein-coding regions of human OR2M3 (NCBI reference sequence: NM_001004689.1), OR2W1 (NCBI reference sequence: NM_030903.3), and OR1A1 (NCBI reference sequence: NM_014565.2) were amplified from human genomic DNA by polymerase chain reaction (PCR) using gene-specific primers (Table S1), ligated with T4-DNA ligase (#M1804, Promega, Madison, USA) either MfeI/NotI for OR2M3 and OR1A1 (#R0589S/#R0189S, New England BioLabs, Ipswich, USA) or *Eco*RI/NotI for OR2W1 (#R6017/#R6435, Promega, Madison, USA) into the expression plasmid pI2-dk(39aa-rho-tag) (aa, amino acid) [53, 54], and verified by Sanger sequencing (Eurofins Genomics, Ebersberg, Germany).

### PCR-based site-directed mutagenesis

All receptor variants used were generated by PCR-based site-directed mutagenesis in two steps. Gene-specific primers (mutation primers) were used according to Table S2 and Table S3. The mutation primers, which carried the changed nucleotides, were designed overlapping.

Step one PCR was carried out in two PCR amplifications, one with the forward gene-specific primer and the reverse mutation-primer, the other with the forward mutation-primer and the reverse gene-specific or vector-internal primer.

Both PCR amplicons were then purified and used as template for step two. Here, the two overlapping amplicons were annealed using the following program: denaturation (98 °C, 3 min), ten cycles containing denaturation (98 °C, 30 s), annealing (start 58 °C, 30 s), and extension (72 °C, 2 min). After this, full-length gene-specific forward and reverse primers were added. The amplicons were then sub-cloned as described above.

### Sequencing

All sub-cloned wild-type (wt) and mutated OR-coding amplicons were verified by Sanger sequencing (Eurofins Genomics, Ebersberg, Germany) using vector-internal primers (Table S4).

### Cell culture and transient DNA transfection

We used NxG 108CC15 cells [55], a neuroblastoma x glioma hybrid, and HEK-293 cells [56], a human embryonic kidney cell-line, as a test cell system for the functional expression of recombinant OR [52].

NxG 108CC15 cells were cultivated at 37 °C, 7% CO_2_, and 100% humidity in 4.5 g/L d-glucose containing DMEM with 10% fetal bovine serum, 4 mmol/L l-glutamine, 100 U/mL penicillin, and 100 U/mL streptomycin, 100 µmol/L hypoxanthine, 0.4 µmol/L aminopterin, 16 µmol/L thymidine (HAT media supplement), and 1% of 100 × MEM non-essential amino acid solution (NEAA). HEK-293 cells were cultivated at 37 °C, 5% CO_2_, and 100% humidity in 4.5 g/L d-glucose containing DMEM with 10% fetal bovine serum, 2 mM l-glutamine, 100 U/mL penicillin, and 100 U/mL streptomycin.

For experiments, cells were plated in a 96-well format (white 96-well plate, Nunc, Roskilde, Denmark) with a density of 7500 cells per well for NxG 108CC15 cells and 12,000 cells per well for HEK-293 cells. On the next day, the transfection was performed by using the lipofection method with each 100 ng/well of the corresponding plasmid DNA as well as with 50 ng/well of the transport protein RTP1S [57], G protein subunit Gαolf [54, 58], olfactory G protein subunit Gγ13 [59], and the pGloSensor™-22F [60] (Promega, Madison, USA) using Lipofectamine^®^ 2000 (#11668-027, Life Technologies, USA). The pGloSensor™-22F is a genetically engineered luciferase with a cAMP-binding pocket, which allows measuring a direct cAMP-dependent luminescence signal. As a control the transfection was performed with the vector plasmid pI2-dk(39aa rho-tag) (aa, amino acids) [53, 54] which is lacking the coding information of an OR together with Gαolf, RTP1S, Gγ13, and cAMP-luciferase pGloSensor™-22F (mock). The amount of transfected plasmid DNA was equal in OR-transfected and mock-transfected cells.

### Luminescence assay

Luminescence assays were performed 42 h post-transfection as reported previously [52]. For experiments without copper, the cells were loaded with a physiological salt buffer (pH 7.5) containing 140 mmol/L NaCl, 10 mmol/L HEPES, 5 mmol/L KCl, 1 mmol/L CaCl_2_, 10 mmol/L glucose, and 2% of beetle luciferin sodium salt (Promega, Madison, USA). For the luminescence measurements, the Glomax^®^ MULTI + detection system (Promega, Madison, USA) was used. After an incubation of the cells for 1 h in the dark, the basal luminescence signal of each well was recorded. Afterwards, the odorant, serially diluted in the physiological salt buffer, was applied to the cells. Odorant stock solutions were prepared in DMSO and diluted 1:1000 in the physiological salt buffer to obtain a final DMSO concentration of 0.1% DMSO on the cells. To keep all measurement conditions the same, the water-soluble substances were also dissolved in DMSO. For odorants which were only slightly soluble, we added Pluronic PE-10500 (BASF, Ludwigshafen, Germany) to the buffer. The final Pluronic PE-10500 concentration on the cells was 0.05%.

Real-time luminescence signals for each well were measured 4 min after the odorant application.

For the measurements with copper, we added a final concentration of 10 µmol/L of a 10 mmol/L CuCl_2_ solution to the normal measurement buffer as described above. The assay was performed as reported above.

### Data analysis of the cAMP-luminescence measurements

The raw luminescence data obtained from the Glomax^®^ MULTI + detection system were analyzed using Instinct Software (Promega, USA). Data points of basal level and data points after odorant application were each averaged. From each luminescence signal, the corresponding basal level was subtracted.

For concentration–response relations, the baseline-corrected data set was normalized to the maximum amplitude of the reference odorant–receptor pair. The data set for the mock control was subtracted and EC_50_ values and curves were derived from fitting the function

$$f\left( x \right) = \left( {\frac{{\left( {min - max} \right)}}{{\left( {1 + \left( {\frac{x}{{EC_{50} }}} \right)^{Hillslope} } \right)}}} \right) + max$$

[61] to the data by nonlinear regression (SigmaPlot 10.0, Systat Software). All data are presented as mean ± SD.

### Homology modeling and docking

Details on the homology model approach for OR2M3 and OR2W1: we used the default setting in MPI Bioinformatics Toolkit server (https://toolkit.tuebingen.mpg.de/#/tools/hhpred), which uses the Modeller program [62] to model the homology model. We built the homology model of OR2M3 using the X-ray structure of the M1 muscarinic receptor as a template (5CXV.pdb) [63]. The comparative protein modeling with available X-ray structures indicates a high sequence identity between the OR2M3 and the M1 receptor transmembrane helix regions. Figure S1 shows the sequence alignment of the human M1 muscarinic receptor (green) and human olfactory receptor OR2M3 (red) as obtained using the Multiple Sequence Viewer implemented in Maestro (Schrödinger Release 2016-3: Maestro, Schrödinger, LLC, New York, NY, 2016.). The TMH domains were obtained using the transmembrane hidden Markov Model (TMHMM) analysis, as applied to model OR5AN1 [64] and OR2T11 [31], using the TMHMM server (http://www.cbs.dtu.dk/services/TMHMM/) based on Bayesian analysis of a pool of transmembrane proteins with resolved structures. As shown in Figure S2, OR2M3 residues with a posterior TMH probability greater than 0.2 were assigned to the transmembrane domain. Similarly, the homology model of OR2W1 was built using the same template (5CXV.pdb), and the TMH regions were obtained by TMHMM analysis. Figure S3 shows the superposition of structures corresponding to the sequence alignment of TMH regions of OR2M3 (red) and OR2W1 (blue) with the human M1 muscarinic receptor (green). As shown in Figure S4, OR2W1 residues with a posterior TMH probability greater than 0.2 were assigned to the transmembrane domain.

*Docking setup* All docking calculations were carried out in the Schrödinger Suite (Small-Molecule Drug Discovery Suite 2016-3, Schrödinger, LLC, New York, NY, 2016.). The initial coordinates of the homology model of the OR2W1 structure were obtained from the homology model as described in the homology model section. Glide SP (standard precision) protocol implemented in Schrödinger Suite was applied for docking (Schrödinger Release 2016-3: Glide, Schrödinger, LLC, New York, NY, 2016.). The receptor was checked for steric clashes as well as for correct protonation states in the protein. The protonation states of all titratable residues (pH = 7) are assigned using PROPKA calculations [65, 66] implemented in the Schrodinger’s Maestro v.9.3 software package (Schrödinger Release 2016-3: Maestro, Schrödinger, LLC, New York, NY, 2016.) and also by visual inspection. Then the receptor was optimized by applying the OPLS_2005 force field [67]. The ligand was docked into the OR2W1 homology model using the GLIDE module [68–70] implemented in the Schrodinger’s Maestro v.9.3 software package. GlideScore (standard precision) was used to rank the different ligands. GlideScore is an empirical scoring function that guesses the ligand-binding free energy. It includes force field (electrostatic and van der Waals) contributions and terms rewarding or penalizing interactions known to influence ligand binding. As it simulates a binding free energy, more negative values represent tighter binders.

### Molecular dynamic simulation

Molecular dynamic simulations were carried out using the CHARMM36 force field implemented in the NAMD2 software [71]. The best docked pose of 3-mercaptohexyl acetate was used as the initial structure. The docking method is described in the docking section. The initial model system was inserted into a water box. After equilibration, production run MD simulations were carried out for 2 ns within the NPT ensemble at 298 K and 1.0 atm using the Langevin piston for 62 ns simulation time for the system (Fig. S5). Electrostatic interactions were treated with the Particle Mesh Ewald (PME) method and van der Waals interactions were calculated using a switching distance of 10 Å and a cutoff of 12 Å. The time step integration was set to 1 fs.

### QM/MM calculations

QM/MM calculations were performed on homology models using ONIOM method [72] as part of the Gaussian 09 software package [73]. The QM layer included the ligand, copper (if present), the copper/ligand-binding residues, and any waters in the binding pocket. For OR2M3, the QM residues were C202, C203, and T105 in site 1 and M118, C241, and H244 in site 2. DFT/M06-L [74, 75], and multiple basis sets were used to describe the QM layer. The 6-31G(d) basis set [76] was applied to carbon, hydrogen, nitrogen, sulfur, and oxygen atoms, and the Stuttgart 8s7p6d2f and 6s5p3d2f ECP10MWB contracted pseudopotential basis set [77] was applied to the copper atom. The MM layer consisted of the remaining protein and was modeled with the AMBER96 force field [78].

### Phylogenetic analysis

For sequence comparison, we used CLC Main Workbench 6.5. We used the same software to perform the ClustalW alignment of the transmembrane regions (TMH) 1–7 and the extracellular loop 2 of all human ORs, family 2 ORs and OR2M3, and 46 homolog receptors as well as 60 orthologs from mouse, rat, chimp, and dog of the three family 2 OR subfamilies M, T, and V (Table S5). We created sequence logos using WebLogo 2.8.2 [79, 80]. The localization of the TMHs of human OR2M3 and OR2W1 was taken from HORDE [81]. The evolutionary history of ORs was inferred using the Neighbor-Joining method [82]. Trees are drawn to scale, with branch lengths in the same units as those of the evolutionary distances used to infer each phylogenetic tree. The evolutionary distances were computed using the Poisson correction method [83] and are in the units of the number of amino acid substitutions per site. All evolutionary analyses were conducted in MEGA7 [84].

## Results

### Copper ions enhanced a thiol agonist action on narrowly tuned OR2M3 but not on broadly tuned OR2W1

**Fig. 1:**
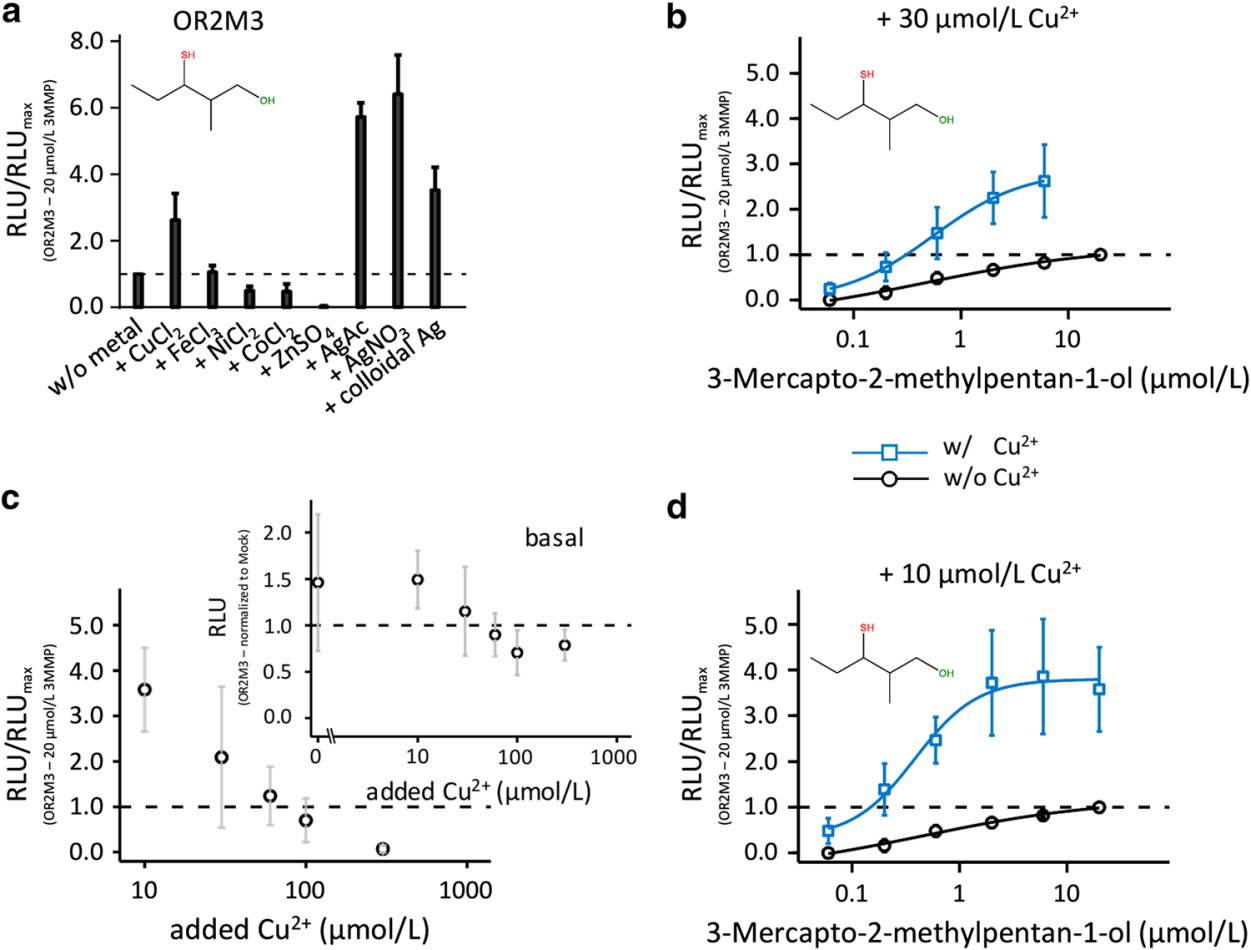
Cu^2+^, Ag^+^, and colloidal silver enhance the efficacy of 3-mercapto-2-methylpentan-1-ol on OR2M3. **a** Comparison of efficacies of 20 µmol/L 3-mercapto-2-methylpentan-1-ol on OR2M3 in the absence or presence of different metal ions at 30 µmol/L; **b** concentration–response relation of 3-mercapto-2-methylpentan-1-ol on OR2M3 in the absence or presence of 30 µmol/L Cu^2+^; **c** effect of 20 µmol/L 3-mercapto-2-methylpentan-1-ol on OR2M3 in the presence of five different Cu^2+^ concentrations and effect of five different copper concentrations on the OR2M3 wt basal levels compared to the buffer without a copper supplementation (data were normalized to the mock basal level and shown as mean ± SD (*n* = 5–10); **d** concentration–response relation of 3-mercapto-2-methylpentan-1-ol on OR2M3 in the absence or presence of 10 µmol/L Cu^2+^. Note that the same data set in the absence of supplemental Cu^2+^ (black) is given in sub-panels (**b**) and (**d**) for didactic reasons. Data were mock control-subtracted, normalized to the OR2M3 wt signal in response to 3-mercapto-2-methylpentan-1-ol (20 µmol/L), measured in the absence of Cu^2+^, and shown as mean ± SD (*n* = 3–6). *RLU*  relative luminescence unit, *3MMP*  3-mercapto-2-methylpentan-1-ol. The concentration–response relation Fig. 1b and Fig. S6a as well as Fig. 1d and Fig. S7a are the identical data set. Also Fig. 1a and Fig. S6 k as well as Fig. 1c and Fig. S7f are the identical data set

Thiols are among the best KFO agonists of the two broadly tuned human odorant receptors OR1A1 and OR2W1 [5], whereas OR2M3 has recently been demonstrated to specifically respond to only 3-mercapto-2-methylpentan-1-ol out of some 190 KFOs [6]. However, the influence of transition metal ions on these thiol/receptor interactions has not been tested so far. We, therefore, examined the effects of different metal ions at a final concentration of 30 µmol/L on the activation of OR2M3 by its agonist 3-mercapto-2-methylpentan-1-ol, an important KFO in heated onions [6], using the cAMP-dependent luminescence-based GloSensor™ assay (Fig. 1a, b, Fig. S6a, Table S6).

In the presence of 30 µmol/L supplemented Cu^2+^, we observed more than 2.5-fold higher amplitudes in response to 3-mercapto-2-methylpentan-1-ol (Fig. 1a, b, Fig. S6a, Table S6). The presence of the copper chelator TEPA at 30 µmol/L not only prevented a Cu^2+^ potentiation, but largely reduced the ligand response of OR2M3 below normal conditions without Cu^2+^ supplementation (Fig. S6b), suggesting that residual Cu^2+^ was present in the commercial bath solution. Without Cu^2+^ supplementation, however, our method could not resolve any ligand-induced amplitudes of OR2T11 (Fig. S6c), suggesting that any residual Cu^2+^ in the commercial bath solution was insufficient for a potentiating effect on ligand-induced signaling of OR2T11 (see also [31]). Fe^3+^ was without effect (Fig. 1a, Fig. S6d, Table S6), but the presence of Ni^2+^ or Co^2+^ reduced the response to 3-mercapto-2-methylpentan-1-ol by half (Fig. 1a, Fig. S6e, f, Table S6). We observed a complete loss-of-function in the presence of Zn^2+^ (Fig. 1a, Fig. S6 g, Table S6). We observed the largest potentiating effects under supplementation with ionic or colloidal silver, which led to a three-to-sixfold increase in amplitude for OR2M3 and 3-mercapto-2-methylpentan-1-ol (Fig. 1a, Fig. S6 h-j, Table S6), suggesting particularly strong ligand/sulfur–silver interactions within the receptor.

We next tested for the optimal Cu^2+^ concentration. As originally suggested by Crabtree [27], the active form of copper involved in ligand coordination is likely Cu^1+^ due to the naturally reducing environment in cells. Therefore, Cu^2+^ added in our experiments was likely reduced to Cu^1+^. We found that adding Cu^2+^ at a concentration of 10 µmol/L gave the highest potentiation of 3-mercapto-2-methylpentan-1-ol´s efficacy in activating OR2M3, which was about fourfold increased as compared to control conditions without Cu^2+^ supplementation (Fig. 1c, d, Fig. S7, Table S7). Importantly, supplementation with Cu^2+^ > 10 µmol/L decreased both 3-mercapto-2-methylpentan-1-ol-induced receptor signaling as well as odorant-independent constitutive activity of OR2M3 (Fig. 1c). Cu^2+^ supplementation of 10 µmol/L, however, did not inhibit a basal activity of OR2M3 (Fig. 1c), and was, therefore, used in all experiments testing a potentiating effect of copper throughout the present study. Cu^2+^ at 10 µmol/L had little effect on the EC_50_ value of 3-mercapto-2-methylpentan-1-ol on OR2M3 (Table 1). Notably, the receptor/agonist pair OR2M3/3-mercapto-2-methylpentan-1-ol when measured in the absence of copper showed a Hill coefficient of 0.89 ± 0.41 (*n* = 4), whereas in the presence of copper (10 µmol/L), the Hill coefficient increased about twofold to 1.93 ± 0.71 (n = 4).

**Table 1:** EC_50_ values for wild-type receptors in the absence and presence of 10 µmol/L Cu^2+^

Receptor	Agonist	EC_50_ in the absence of Cu^2+^ in µmol/L^a^	EC_50_ in the presence of Cu^2+^ in µmol/L^a^
OR2M3 wt	3-Mercapto-2-methylpentan-1-ol	0.45 ± 0.19	0.29 ± 0.10
OR2W1 wt	2-Phenylethanethiol	37.81 ± 0.62	106.21 ± 0.93
OR2W1 wt	3-Mercaptohexyl acetate	132.10 ± 20.86	120.78 ± 1.59
OR2W1wt	Allyl phenyl acetate	64.40 ± 3.58	55.15 ± 8.59
OR1A1 wt	(*R*)-(-)-Carvone	126.47 ± 34.07	159.03 ± 43.09
OR1A1 wt	2-Phenylethanethiol	205.94 ± 21.53	73.06 ± 16.35
OR1A1 wt	3-Mercaptohexyl acetate	404.65 ± 86.73	353.78 ± 113.08
A2A/A2B	Adenosine	471.75 ± 35.10	452.95 ± 38.26
S1P4^b^	CYM50308	516.51 ± 74.57	343.76 ± 91.02

^a^Mean ± SD (*n* = 3–5)

^b^*EC*_50_ in nmol/L

**Fig. 2:**
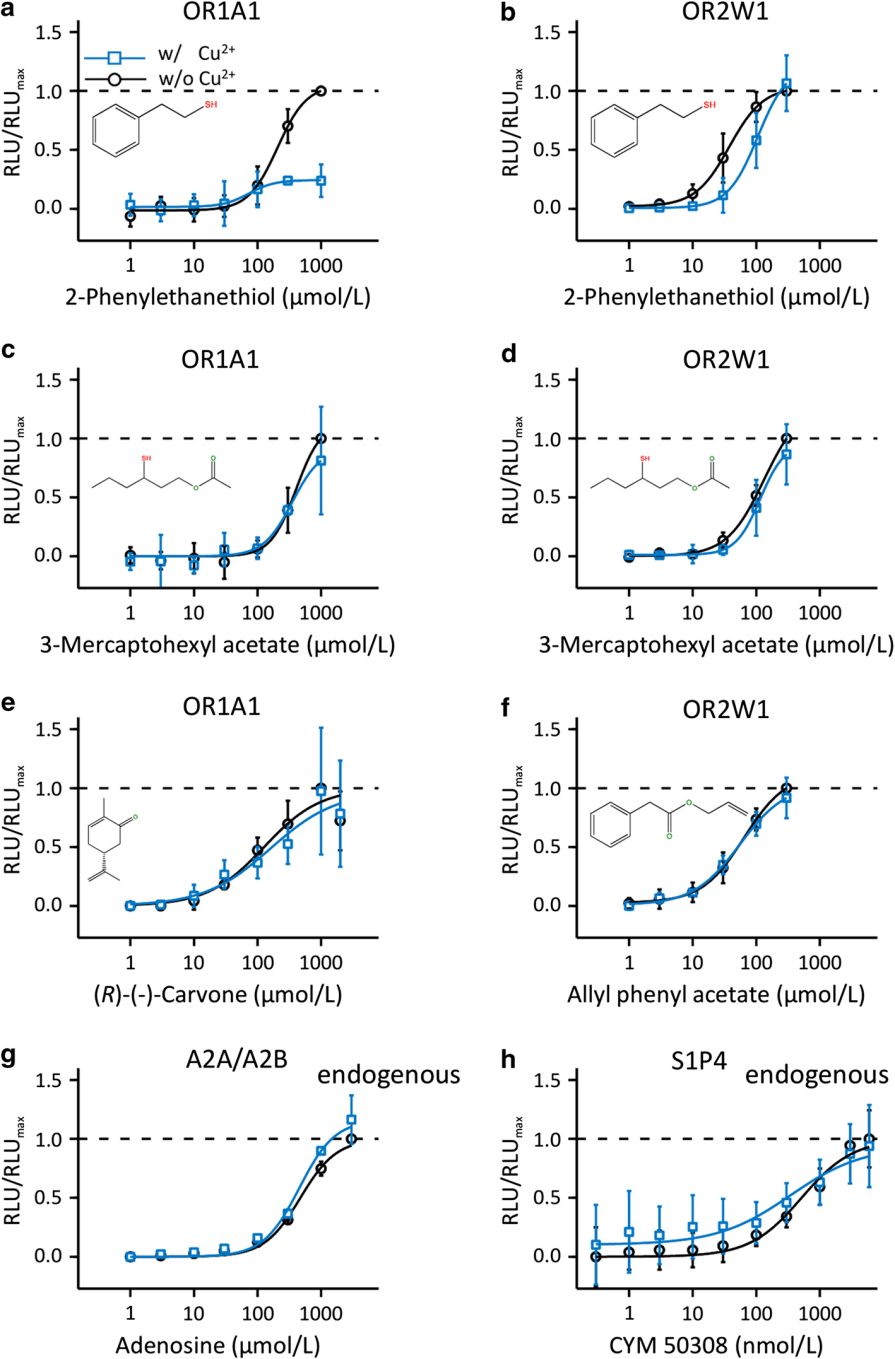
Cu^2+^ diminished the efficacy or potency of 2-phenylethanethiol of broadly tuned OR1A1 and OR2W1, but not of 3-mercaptohexyl acetate or non-thiol receptor agonists. Effects of 10 µmol/L Cu^2+^ on the concentration–response relations of thiol and non-thiol agonists of OR1A1 (**a**, **c**, **e**), OR2W1 (**b**, **d**, **f**), and endogenously expressed GPCRs, adenosine receptors A2A/A2B (**g**), and sphingosine-1-phosphate receptor 4, S1P4 (**h**). Data were mock control-subtracted, normalized to each receptor maximum amplitude as response to the respective substance measured in the absence of Cu^2+^, and shown as mean ± SD (*n* = 3–6). *RLU*  relative luminescence unit. Curves represent best fits to the data in the absence (black) or presence (blue) of Cu^2+^, with EC_50_ values given in Table 1

In contrast to the potentiating effect of Cu^2+^ on OR2M3, the efficacy of 2-phenylethanethiol in activating OR1A1 was reduced in the presence of Cu^2+^ at 10 µmol/L (Fig. 2a, Table 1). Indeed, the *EC*_50_ value of 2-phenylethanethiol on OR2W1 was increased in the presence of Cu^2+^, indicating a lower potency of 2-phenylethanethiol in activating broadly tuned OR2W1 (Fig. 2b, Table 1). Cu^2+^ at 10 µmol/L did not affect the concentration–response relations of two KFO thiol agonists of broadly tuned OR2W1, 2-phenylethanethiol, or 3-mercaptohexyl acetate, or its non-thiol agonist allyl phenyl acetate (Fig. 2b, d, f, Table 1). Likewise, the presence of Cu^2+^ affected neither the activation of broadly tuned OR1A1 by its thiol agonist 3-mercaptohexyl acetate (Fig. 2c, Table 1) nor by (*R*)-(-)-carvone (Fig. 2e, Table 1). Also, the activation of NxG 108CC15-endogenous adenosine receptors A2A/A2B [52] or sphingosine-1-phosphate receptor 4 (S1P4) receptors by their respective agonists was not affected by copper supplementation (Fig. 2g, h, Table 1).

### Cys179 of the HxxC[DE]-Motif in ECL 2 of ORs plays a copper-independent role for a receptor function

**Fig. 3:**
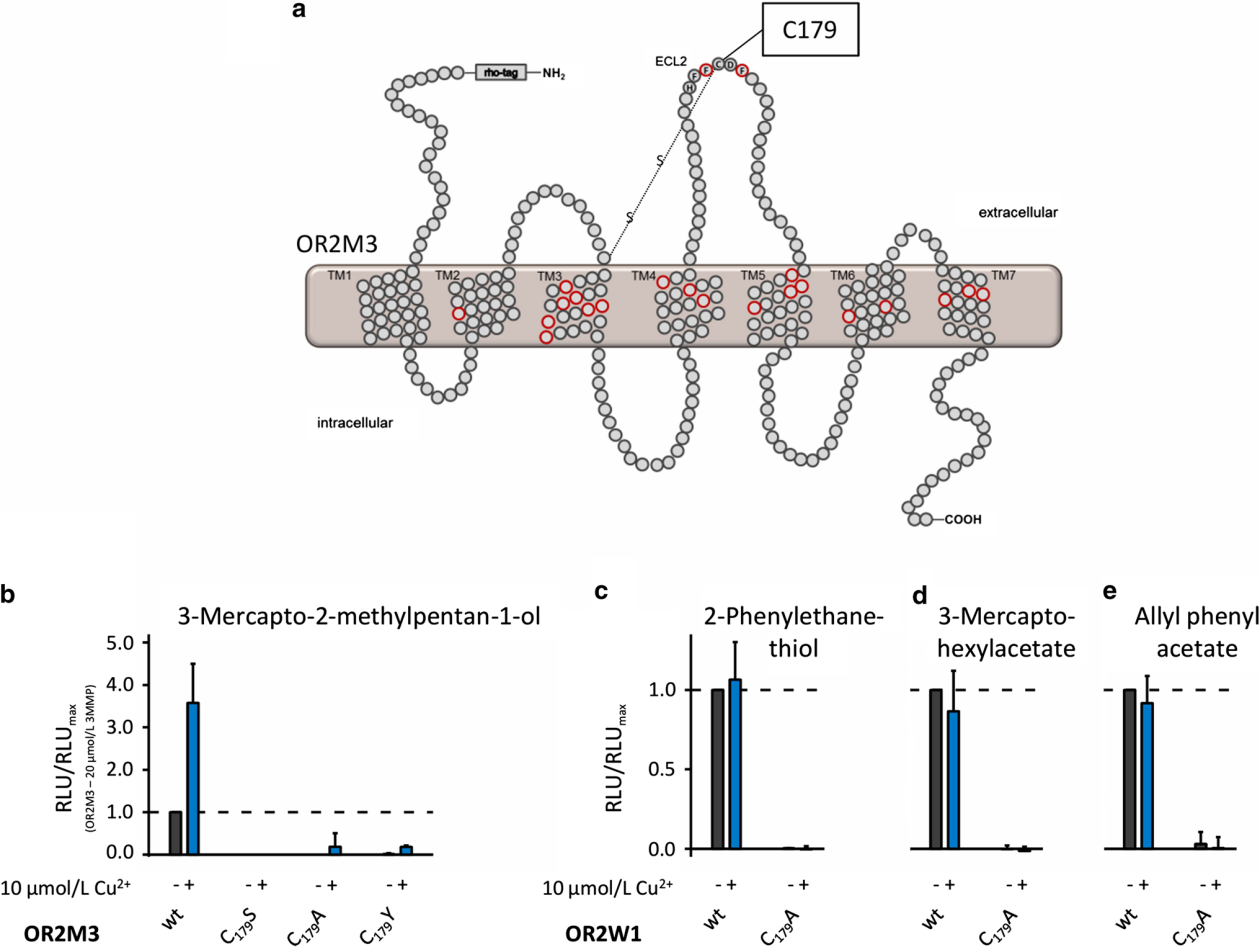
Mutating the conserved cysteine in the HxxC[DE]-motif (Wang et al. [28]) abolishes odorant responses of ORs independent of Cu^2+^. **a** Schematic snake diagram of OR2M3 with localization of amino acid position Cys179 within the HxxC[DE]-motif of ECL 2. Putative odorant interaction sites proposed by Man et al. [85] are given as red circles. **b** Effects of different Cys179 mutations in OR2M3, tested with 3-mercapto-2-methylpentan-1-ol (20 µmol/L), and compared to the wild-type (wt) receptor. (**c–e**) Effects of thiol and non-thiol agonists (300 µmol/L, each) on OR2W1 C_179_A. Data were recorded in the absence (black) or presence (blue) of Cu^2+^, normalized to the respective wt signal in the absence of Cu^2+^, and represented as mean ± SD (*n* = 3). *RLU*  relative luminescence unit, *3MMP*  3-mercapto-2-methylpentan-1-ol

To challenge the hypothesis of a metal-coordinating role of the HxxC[DE] motif of ECL 2 in the majority of ORs posed by Wang et al. [28], we established variants of OR2M3 and OR2W1, changing the conserved cysteine at position 179 to an alanine by site-directed mutagenesis. For OR2M3, we further established the variant OR2M3 C_179_Y, which was described as single-nucleotide polymorphism (SNP) [85], and a variant where we changed the cysteine to a serine. Already in the absence of any copper supplementation, all of these receptor variants displayed a complete loss-of function when tested with their respective agonists (Fig. 3b–e), suggesting a rather general role of at least Cys179 for the tertiary structure of ORs [86–88].

### TMH 5 and TMH 6 of OR2M3 harbor cysteines necessary for a potentiating effect of Cu^2+^ on 3-mercapto-2-methylpentan-1-ol function in OR2M3

Sekharan et al. [30] showed with their QM/MM model and mutagenesis-guided functional experiments that copper supposedly binds to His105^3.33^, Cys109^3.37^, and Asn202^5.42^ in an internal aqueous channel of Olfr1509 (MOR244-3). They proposed Cys109^3.37^ of Olfr1509 to coordinate the copper ion in the receptor [30]. Based on the QM/MM model by Sekharan et al. [30], we prepared variants of OR2M3 by changing the respective amino acids of OR2M3 to the amino acids of Olfr1509 at the positions 105^3.33^ and 109^3.37^. Moreover, we prepared SNP-based haplotypes: For position 105^3.33^, we changed the threonine to an isoleucine or to an alanine. Both, however, have a minor allele frequency (MAF) of only 0.008 [89]. Furthermore, we changed the glycine at position 109^3.37^ to an arginine [85]. Since cysteine contains an S atom and is a polar amino acid, its free thiol group can build S–S bonds after oxidation. Furthermore, the binding of copper or other metal ions such as zinc and iron often occurs at cysteine residues in metalloproteins [90–93]. Therefore, and since Cys112^3.40^ is in the vicinity of positions 105^3.33^ and 109^3.37^, we exchanged Cys112^3.40^ to a serine or an alanine.

**Fig. 4:**
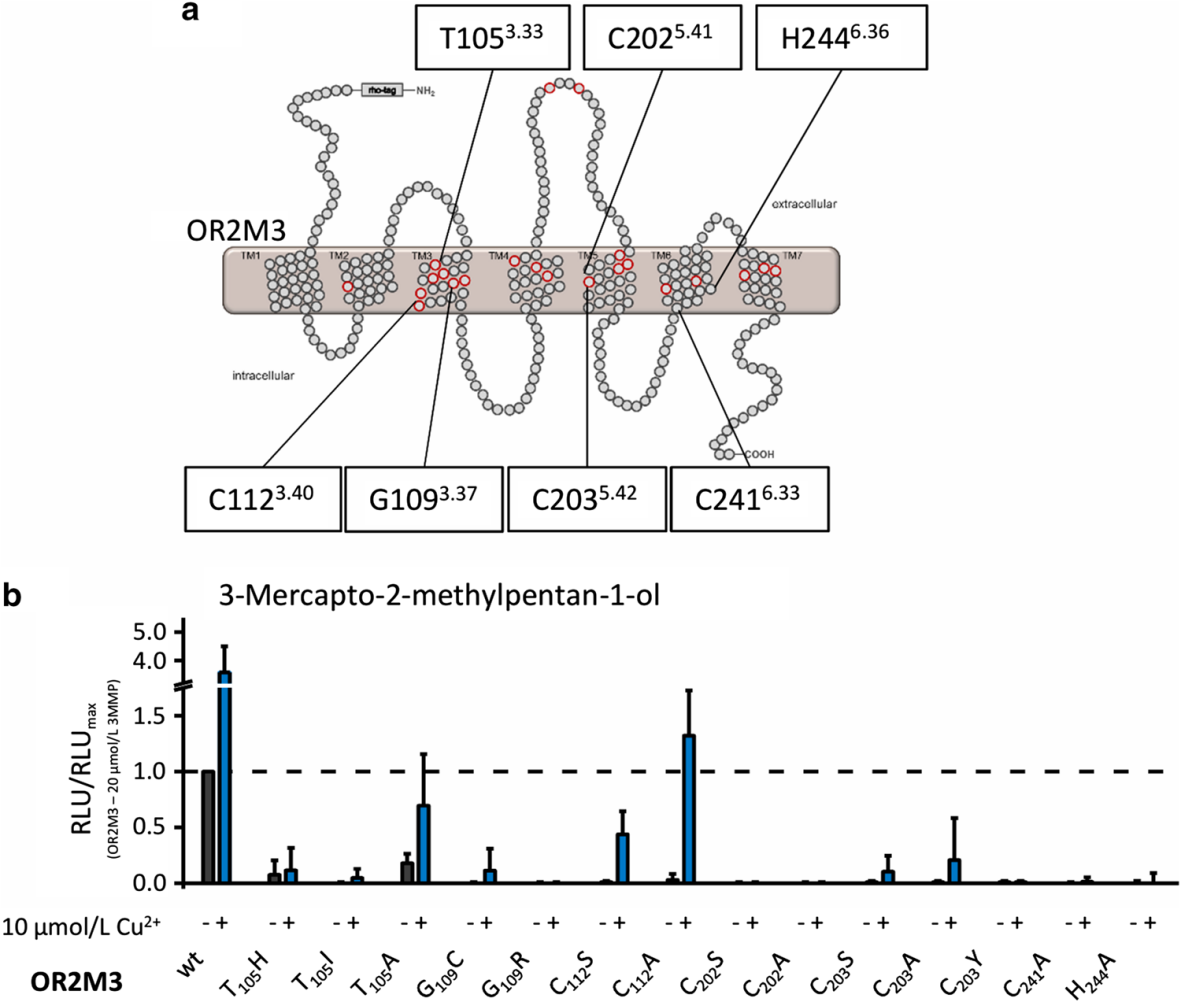
Testing amino acid positions of proposed copper/odorant-binding pockets [30–32] by site-directed mutagenesis in OR2M3. **a** Schematic snake diagram of OR2M3 with localization of mutated amino acid positions within TMH 3-6. Putative odorant interaction sites proposed by Man et al. [85] are given as red circles. **b** Effect of 3-mercapto-2-methylpentan-1-ol (20 µmol/L) on OR2M3 mutants, in the absence (black) or presence (blue) of Cu^2+^. Data were normalized to the OR2M3 wt signal in response to 3-mercapto-2-methyl-pentan-1-ol (20 µmol/L), measured in the absence of Cu^2+^. Shown are mean ± SD (*n* = 3). *RLU* relative luminescence unit, *3MMP* 3-mercapto-2-methylpentan-1-ol. Concentration–response curves for all mutant receptors are given in Supplemental Figure S8, and EC_50_ values are given in Table 2

We found that all OR variants with mutations at position 105^3.33^, 109^3.37^, or 112^3.40^ were not functional anymore (Fig. 4b). However, the presence of copper 10 µmol/L rescued 3-mercapto-2-methylpentan-1-ol function in OR2M3, at least for mutations T_105_A, C_112_A, and C_112_S (Fig. 4b, Fig S8a, b, and Table 2). This suggested that other positions in OR2M3 were involved in a copper interaction.

**Table 2:** *EC*_50_ values for OR2M3 with amino acid changes at positions of a putative copper/odorant-binding pocket measured in the absence and presence of 10 µmol/L Cu^2+^

Receptor	Agonist	EC_50_ in the absence of Cu^2+^ in µmol/L^a^	EC_50_ in the presence of Cu^2+^ in µmol/L^a^
OR2M3 T_105_A	3-Mercapto-2-methylpentan-1-ol	1.42 ± 0.58	0.75 ± 0.27
OR2M3 C_112_A	3-Mercapto-2-methylpentan-1-ol	n.d.	0.86 ± 0.004
OR2M3 C_203_S	3-Mercapto-2-methylpentan-1-ol	n.d.	3.16 ± 0.01

*n.d.* no detectable response up to 100 µmol/L

^a^Mean ± SD (*n* = 3–5)

Zhang et al. [32] showed for the murine receptor Olfr1019 that Cys203^5.42^ is involved in binding copper.

OR2M3 contains two adjacent cysteines at positions 202^5.41^ and 203^5.42^. We, therefore, changed these cysteines to either an alanine or to a serine. OR2M3 variants carrying mutations C_202_A, C_202_S, or C_203_A revealed a complete loss-of-function, even in the presence of Cu^2+^, with a strongly diminished 3-mercapto-2-methylpentan-1-ol function in C_203_A (Fig. 4b, Fig. S8c, and Table 2).

We further investigated a putative functional role of the cysteines at positions 202^5.41^ and 203^5.42^ in OR2M3. Indeed, at least position 203^5.42^ aligns with a putative odorant-binding pocket suggested by Man et al. [88]. Furthermore, we investigated the haplotype with one SNP, OR2M3 C_203_Y, which has an MAF of < 0.01 [89]. In our hands, OR2M3 C_203_Y showed a complete loss-of-function, in the absence or presence of Cu^2+^. Altogether, our results suggest both positions Cys202^5.41^ and Cys203^5.42^ to be necessary for a potentiating effect of Cu^2+^ on a 3-mercapto-2-methylpentan-1-ol function in OR2M3.

Our concentration–response relation of 3-mercapto-2-methylpentan-1-ol on OR2M3 wt in the presence of Cu^2+^ revealed a Hill coefficient close to 2 (see Fig. 1d), suggesting positive cooperativity of at least two binding sites for Cu^2+^ and/or 3-mercapto-2-methylpentan-1-ol. Indeed, within copper-dependent human OR2T11, two distinct copper-binding sites have been reported previously, constituted by positions Met56^2.39^, Met133^4.37^, Arg135^4.39^, and Cys138^4.42^, and positions Met115^3.46^, Cys238^6.33^, and His241^6.36^ [31]. Positions Cys238^6.33^ and His241^6.36^ in OR2T11 correspond to positions Cys241^6.33^ and His244^6.36^ in OR2M3, respectively. Since positions Cys238^6.33^ and His241^6.36^ are part of the putative copper-binding CSSH(L) motif in OR2T11, which is close to the cytoplasmic region, similar to other candidate pentapeptides previously proposed for metal-binding sites at the end of TMH 6 [34], we mutated the corresponding positions Cys241^6.33^ and His244^6.36^ in OR2M3 by changing the respective amino acids to an alanine. In our hands, both OR2M3 variants were not functional anymore, in the absence or presence of Cu^2+^ (Fig. 4b), suggesting both positions to be necessary for a potentiating effect of Cu^2+^ on a 3-mercapto-2-methylpentan-1-ol function in OR2M3.

### SNPs in close vicinity of predicted copper/odorant-binding positions affected the 3-mercapto-2-methylpentan-1-ol function of OR2M3

Previously, Man et al. [88] determined 22 amino acid positions, which constitute a putative, generalized, and conserved odorant-binding pocket within OR orthologs. We, therefore, investigated the effects of three SNPs that occur in the putative binding pocket of OR2M3 near proposed copper-interacting positions [30], by testing these haplotypes against 3-mercapto-2-methylpentan-1-ol in the GloSensor™ assay (Fig. S9). For the variant, OR2M3 M_206_I, we observed a complete loss-of-function (Fig. S9c). Compared to OR2M3 wt, OR2M3 Y_104_C displayed a gain of function with respect to the amplitude but a higher *EC*_50_ value (1.02 ± 0.18 µmol/L, Fig. S9b), whereas OR2M3 I_207_L had a diminished amplitude and a higher *EC*_50_ value (1.41 ± 0.04, Fig. S9d). All SNPs in OR2M3, however, have either no reported MAF, or a rather low MAF (< 0.01).

### Homology modeling and QM/MM studies revealed two copper-coordinating sites within narrowly tuned OR2M3

**Fig. 5:**
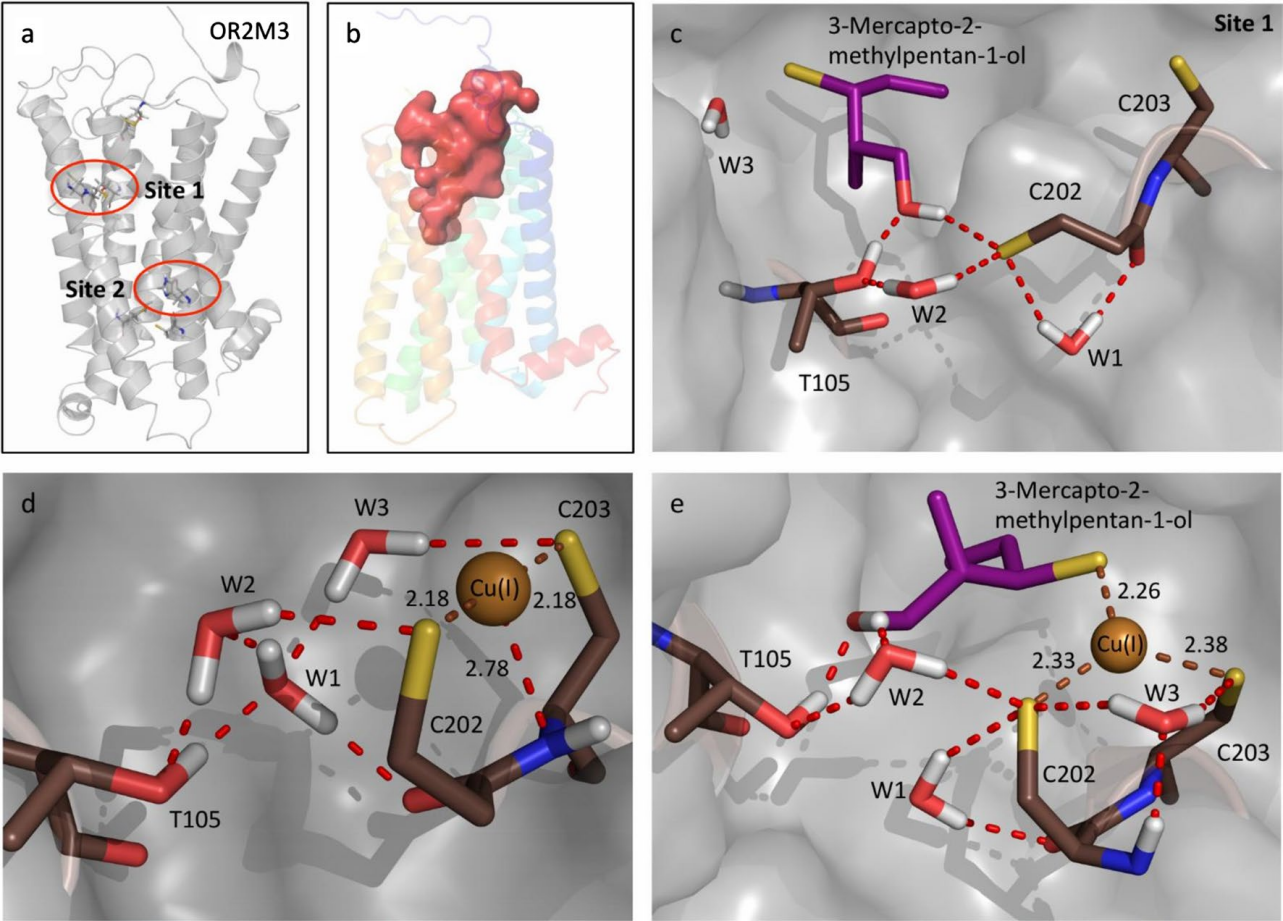
Molecular modeling reveals two 3-mercapto-2-methylpentan-1-ol/copper-binding sites within OR2M3. **a** Binding sites within the homology model of OR2M3, involving TMH 3 and TMH 5 (site 1) or TMH 3 and TMH 6 (site 2). **b** Accessible volume of the ligand in OR2M3 (site 1). QM/MM structural model of site 1 in OR2M3 with the ligand 3-mercapto-2-methylpentan-1-ol (**c**), with Cu(I) (**d**), or both (**e**). Residues defining the binding pocket are shown as sticks (oxygen: red; nitrogen: blue; sulfur: yellow)

Figure 5a shows the structural model of OR2M3 obtained using the X-ray crystal structure of the human M1 muscarinic receptor as a template (5CXV.pdb) [63]. The homology model provides valuable insights on the proposed odorant-binding site, including a highly conserved disulfide S–S bond thought to be critical for structural stability. The structural model of OR2M3 shows that the disulfide bond forms between Cys97^3.25^ of TMH 3 and Cys179 of extracellular loop 2 (ECL 2) (Fig. 5a). Two binding sites for Cu(I) were identified in OR2M3 (Fig. 5, Fig. S10). The binding sites are supported by site-directed mutagenesis and activation profiles, showing a lack of response to ligand when mutating the key amino acid residues responsible for copper binding (Fig. S10).

The first ligand/copper-binding site (site 1) shares similarities with the copper-binding site suggested for Olfr1509 (MOR244-3) [30] with one Cu(I) bound to His105^3.33^, Cys109^3.37^, and Asn202^5.42^; the copper-binding site in Olfr1509 is close to the extracellular domain. In contrast, OR2M3 involves two binding sites. Site 1 has an accessible volume of 695.26 Å^3^ for 3-mercapto-2-methylpentan-1-ol (Fig. 5b), and involves Thr105^3.33^ of TMH 3, and residues Cys202^5.41^ and Cys203^5.42^ from TMH 5 (Fig. 5c). The Cu(I)_S1_ ion has a trigonal planar configuration with S_C202_, S_C203_ and a weak interaction with N_C203_, with distances of 2.18 Å, 2.18 Å and 2.78 Å, respectively, as shown in Fig. 5d, which indicates the QM/MM structural model for OR2M3. It is also important to note that water molecules form hydrogen bonds with residues Thr105^3.33^, Cys202^5.41^, and Cys203^5.42^. For example, the water molecule (W1) forms hydrogen bonds O_C202_-HO_W1_ and OH_T105_-O_W1_ with distances of 1.88 Å and 1.79 Å, respectively. Another water molecule (W2) is also located between residues Thr105^3.33^ and Cys202^5.41^, forming hydrogen bonds HO_T105_–HO_W2_ and S_C202_–HO_W2_, with distances of 2.05 Å and 2.31 Å, respectively. HO_W1_–O_W2_ forms an H-bond with a distance of 1.85 Å. The third water molecule (W3) form hydrogen bonds S_C203_–HO_W3_ and O_W1_–HO_W3_ with distances of 2.49 Å and 2.00 Å, respectively.

Upon ligand (3-mercapto-2-methylpentan-1-ol) binding, the active sites undergo coordination rearrangements. Figure 5e shows the QM/MM structure of the binding site with the ligand. At site 1, the Cu(I)_S1_ ion has trigonal planar geometry with the S (thiolate form) of ligand, S_C202_ and S_C203_. The distances between Cu(I)_s1_ and S (thiolate forms) of the ligand, S_C202_ and S_C203_ are 2.26 Å, 2.33 Å, and 2.38 Å, respectively. The distance between the Cu(I)_S1_ ion and N_C203_ elongated to 3.14 Å from 2.78 Å. While the S atom of the ligand coordinates with Cu(I), the ligand O (alcoholic) forms a strong H-bond with the HO of Thr105^3.33^ with the distance of 1.80 Å, which elongates OH_T105_-O_W1_ H-bond. We also find that water molecule W1 forms a new but weak H-bond with Cys202^5.41^, S_C202_-HO_W1_, with a distance of 2.38 Å.

Moreover, HO_ligand_ also shows a strong 1.88 Å H-bond with O_W2_. These new H-bond formations slightly displace water molecule W2 towards residue Cys202^5.41^, with a 2.25 Å S_C202_–HO_W2_ distance. However, the HO_T105_–HO_W2_ distance did not change upon ligand binding. Like W2, W3 also moves towards the residues Cys202^5.41^ and Cys203^5.42^, forming new S_C203_–HO_W3_ and O_W3_–HN_C202_ H-bonds with respective distances of 2.35 Å and 1.99 Å.

The second binding site (site 2) includes residues Met118^3.46^ of TMH 3, His244^6.36^, Cys241^6.33^ of TMH 6, and a water molecule (W4) (Fig. S10a). Unlike site 1, Cu(I)_S2_ ion forms a tetrahedral configuration with S_M118_, S_C241_, N_H244_, and O_W4_ with distances of 2.41 Å, 2.16 Å, 2.00 Å, and 2.36 Å, respectively (Fig. S10b). Water molecule W4 also shows an H-bond (N_C241_-HO_W4_) with a distance of 2.18 Å. Upon ligand binding at site 2, Cu(I)_S2_ accommodates a distorted tetrahedral geometry with S_ligand_, S_C241_, N_H244_, and O_W4_ with distances of 2.29 Å, 2.18 Å, 2.02 Å, and 3.06 Å, respectively (Fig. S8c). The distance of the residue Met118^3.46^ and Cu ion extends to 4.54 Å from 2.41 Å. A strong H-bond is also observed between OH_ligand_ and O_W4_ with a distance of 1.98 Å. In addition, the water W4 shows a weak H-bond interaction with the S (thiolate) of the ligand by the distance of 2.21 Å.

### Modeling did not support evidence for a putative copper-binding site within the broadly tuned OR2W1

**Fig. 6:**
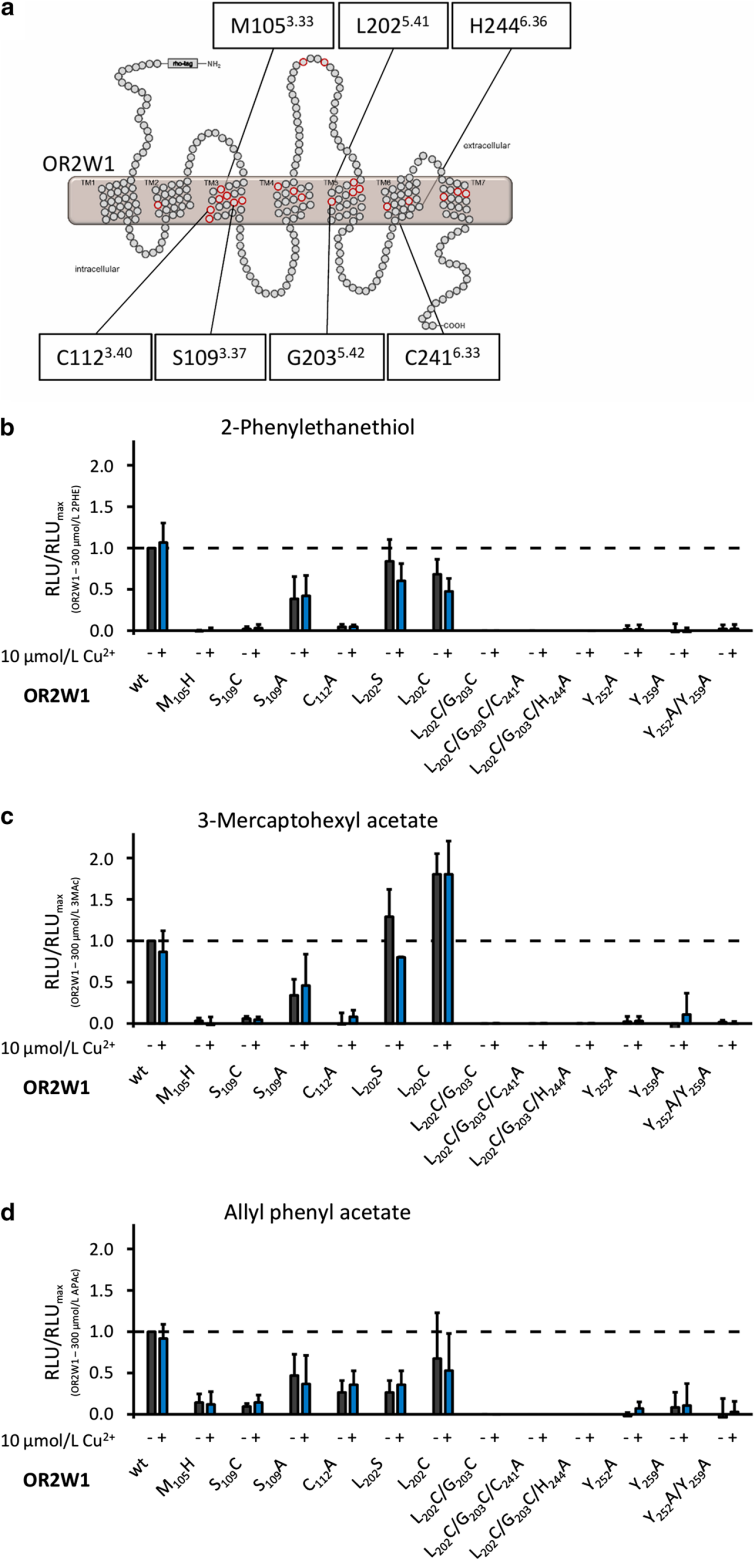
Testing amino acid positions of proposed copper/odorant-binding pockets [30–32] by site-directed mutagenesis in OR2W1. **a** Schematic snake diagram of OR2W1 with localization of mutated amino acid positions within TMH 3–6. Putative odorant interaction sites proposed by Man et al. [85] are given as red circles. Effect of 2-phenylethanethiol (300 µmol/L) (**b**), 3-mercaptohexyl acetate (300 µmol/L) (**c**), and allyl phenyl acetate (300 µmol/L) (**d**) on OR2W1 mutants in the absence (black) or presence (blue) of Cu^2+^. Data were mock control-subtracted, normalized to the OR2W1 wt signal of each ligand, measured in the absence of Cu^2+^, and displayed as mean ± SD (*n* = 3). *RLU*   relative luminescence unit, *2PHE* 2-phenylethanethiol, *3MAc* 3-mercaptohexyl acetate, *APAc* allyl phenyl acetate. Concentration–response curves for all mutant receptors are given in Supplemental Figure S8, and EC_50_ values are given in Table 3

Sekharan et al. [30] proposed Cys109^3.37^ of Olfr1509 to coordinate the copper ion in the receptor [30]. To investigate if we can induce a copper-enhancing effect also in OR2W1, we changed the respective amino acids of OR2W1 to the amino acids of Olfr1509 at the positions 105^3.33^ and 109^3.37^. We performed the point mutation OR2W1 M_105_H, because histidine can act as a ligand of metal ion complexes and possibly can induce a copper-enhancing effect in OR2W1. We observed, however, a loss-of-function with all three tested ligands, suggesting that histidine prevents the formation of the functionally active network of contact sites in OR2W1 (Fig. 6b–d).

We then tried to induce a copper-enhancing effect by introducing a cysteine at position 109^3.37^. Again, we observed a loss-of function with all three tested ligands (Fig. 6b–d). Nevertheless, introducing an alanine at position 109^3.37^, the OR2W1 variant S_109_A was still functional, but displayed agonist-specific and Cu^2+^-dependent differences in potency and efficacy (Fig. 6 b–d, Fig. S8j-l, Table 3). Since the free thiol group of cysteine can build S–S bonds, we further investigated Cys112^3.40^ in OR2W1 and exchanged the cysteine for an alanine. OR2W1 C_112_A did not respond to one of its agonists, in the absence or presence of Cu^2+^ (Fig. 6b–d).

**Table 3:** *EC*_50_ values for OR2W1 with amino acid changes at positions of a putative copper/odorant-binding pocket measured in the absence and presence of 10 µmol/L Cu^2+^

Receptor	Agonist	EC_50_ in the absence of Cu^2+^ in µmol/L^a^	EC_50_ in the presence of Cu^2+^ in µmol/L^a^
OR2W1 L_202_S	2-Phenylethanethiol	97.03 ± 13.01	96.73 ± 4.66
OR2W1 L_202_C	2-Phenylethanethiol	65.08 ± 10.80	63.41 ± 3.77
OR2W1 S_109_A	2-Phenylethanethiol	34.41 ± 9.98	38.93 ± 5.38
OR2W1 L_202_S	3-Mercaptohexyl acetate	146.00 ± 6.48	310.73 ± 22.11
OR2W1 L_202_C	3-Mercaptohexyl acetate	98.89 ± 7.49	114.43 ± 10.46
OR2W1 S_109_A	3-Mercaptohexyl acetate	146.49 ± 23.01	126.95 ± 84.89
OR2W1 L_202_S	Allyl phenyl acetate	37.60 ± 11.68	46.00 ± 14.34
OR2W1 L_202_C	Allyl phenyl acetate	129.92 ± 82.50	73.00 ± 42.12
OR2W1 S_109_A	Allyl phenyl acetate	46.04 ± 11.63	118.90 ± 19.92

*n.d.* no detectable response up to 100 µmol/L

^a^Mean ± SD (*n* = 3–5)

Our results so far suggested that in OR2M3, two cysteines at positions 202^5.41^ and 203^5.42^ coordinate copper in the ligand-binding pocket. Similarly, Cys203^5.42^ has recently been shown to be involved in copper binding in Olfr1019 [32]. OR2W1, however, lacks cysteines at these positions. We, therefore, tested whether cysteines at positions 202^5.41^ and 203^5.42^ will introduce a copper-dependent ligand response in OR2W1. When changing the leucine at position 202^5.41^ to a cysteine or to a serine, in our hands, both OR2W1 variants L_202_S or L_202_C were still functional, but displayed agonist-specific and Cu^2+^-dependent differences in potency and efficacy (Fig. 6b–d).

For 2-phenylethanethiol, both OR2W1 L_202_S and OR2W1 L_202_C displayed lower amplitudes in the presence of Cu^2+^, as compared to OR2W1 wt (Fig. 6b), and a lower potency (Fig. S8d, g, Table 3), although both potency and efficacy of 2-phenylethanethiol were already diminished in the absence of Cu^2+^. For the ‘black currant’-like smelling 3-mercaptohexyl acetate, we observed increased amplitudes compared to OR2W1 wt for both OR2W1 L_202_S and OR2W1 L_202_C (Fig. S8e, h). The *EC*_50_ values for OR2W1 L_202_S were higher as compared to the wild type, with or without supplemental copper, but were lower for OR2W1 L_202_C (Table 3). The non-KFO allyl phenyl acetate revealed concentration–response relations for both OR2W1 Leu202^5.41^ variants, with amplitudes reduced by half as compared to OR2W1 wt (Fig. S8f, i). Under both Cu^2+^ conditions, and compared to OR2W1 wt, the *EC*_50_ values of allyl phenyl acetate for OR2W1 L_202_S were smaller, as observed also for 3-mercaptohexyl acetate, but, however, were higher for OR2W1 L_202_C, as compared to 3-mercaptohexyl acetate (Table 3).

We further inserted the second cysteine at position 203^5.42^ in OR2W1, and tested OR2W1 L_202_C/G_203_C with all three ligands. This variant, however, was not functional anymore (Fig. 6b–d).

Our results additionally identified amino acids Cys241^6.33^ and His244^6.36^ as being necessary for a potentiating effect of copper on 3-mercapto-2-methylpentan-1-ol function in OR2M3. Indeed, these positions previously have been reported to coordinate copper in OR2T11 [31]. We mutated the first and last positions of the CSSH motif in OR2W1, Cys241^6.33^, and His244^6.36^, by changing each amino acid to an alanine, and combined each with the double-mutation OR2W1 L_202_C/G_203_C. In our hands, both OR2W1 variants, OR2W1 L_202_C/G_203_C/C_241_A and OR2W1 L_202_C/G_203_C/H_244_A were not functional anymore for all three tested compounds, in the absence or presence of Cu^2+^ (Fig. 6b–d).

For several odorant receptors, tyrosines at positions 252^6.44^ and 259^6.51^ have been shown to be involved in ligand binding [10, 32, 50, 94–99]. We, therefore, exchanged these tyrosines to alanines and tested the resulting OR2W1 variants against the three agonists 2-phenylethanethiol, 3-mercaptohexyl acetate, and allyl phenyl acetate. Both OR2W1 Y_252_A and OR2W1 Y_259_A, as well as the double mutant OR2W1 Y_252_A/Y_259_A were non-functional (Fig. 6b–d).

### Docking and molecular dynamic simulation of broadly tuned OR2W1 revealed a ligand-binding site about twice as big as in narrowly tuned OR2M3

**Fig. 7:**
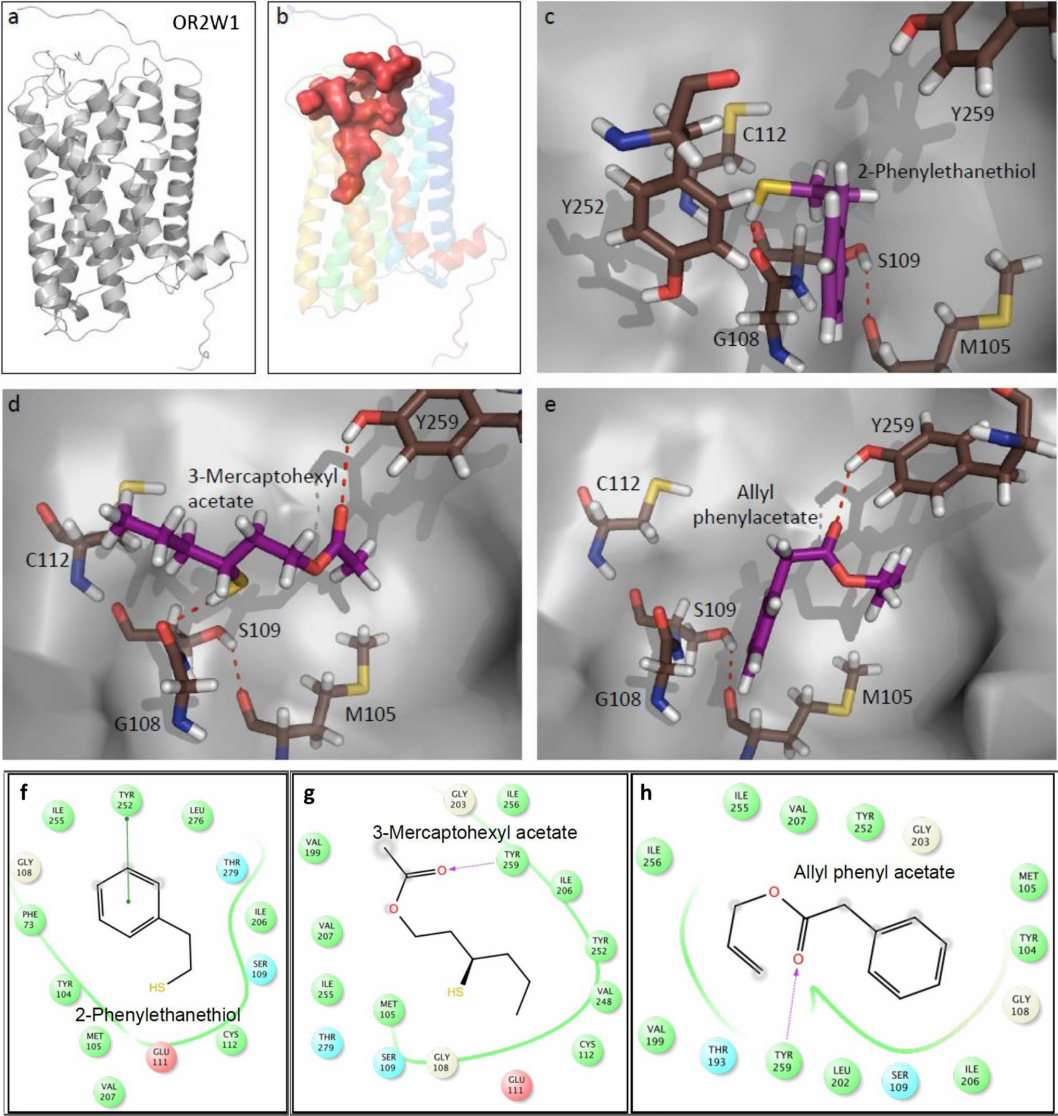
Molecular modeling reveals no copper-binding site within OR2W1. **a** Homology model of OR2W1. **b** Accessible volume of the ligand in OR2W1. QM/MM structural models for OR2W1 show the docked ligands 2-phenylethanethiol **c**, 3-mercaptohexyl acetate (**d)**, or allyl phenyl acetate (**e)**. Residues defining the binding pocket are shown as sticks (oxygen: red; nitrogen: blue; sulfur: yellow). Ligand–OR2W1 interactions for 2-phenylethanethiol (**f**), 3-mercaptohexyl acetate (**g**), or allyl phenyl acetate (**h**). Polar residues (blue), hydrophobic residues (green), negative charged residues (red), Glycine (beige), π–π stacking (green line), and H-bonding interactions (dashed magenta line)

Figure 7a shows the homology model of OR2W1. Figure 7b shows the accessible volume of the ligand for OR2W1 which is 1138.07 Å^3^, compared to 695.26 Å^3^ for OR2M3 (see Fig. 5b). We performed molecular dynamic simulation to see the dynamic stability of ligands in the binding site of OR2W1. The docking calculations show that the ligands, 3-mercaptohexyl acetate and allyl phenyl acetate, bind by forming an H-bond with Tyr259^6.51^ in OR2W1 (Fig. 7c–h). The residue Tyr259^6.51^ is on the top of the TM region and close to the extracellular loop. The 3-mercaptohexyl acetate (Fig. 7d + g) and allyl phenyl acetate (Fig. 7e + h) show similar results. However, 2-phenylethanethiol does not show any H-bond with Tyr259^6.51^, but rather π–π stacking with Tyr252^6.44^ (Fig. 7c + f). The binding site also consists of Met105^3.33^, Ser109^3.37^, and Cys112^3.40^, however, we did not find any H-bonding interactions with the ligand. Rather, these residues appear to be important for stabilizing the ligand-binding site (see Fig. 7c–e, H-bond between Met105^3.33^ and Ser109^3.37^).

It is known that dynamical binding modes determine agonistic and antagonistic ligand effects in GPCRs [100]. Our 62 ns stimulation of OR2W1 in a water box without a membrane revealed a stabilization to ~ 4.5 Å after the first 12 ns (Fig. S5), and for the ligand 3-mercaptohexyl acetate, a free-binding energy of − 22.67 ± 2.02 kcal/mol. In the last 50 ns of the simulation, the ligand hydrogen bonded with the Tyr259^6.51^ side chain 64.33% of the time, the Val199^5.38^ backbone 48.34%, and Gly203^5.42^ only 0.16%. Thus, dynamic modeling confirmed Tyr259^6.51^ as the major ligand-interaction site via an H-bond as proposed by our static model.

### The putative copper-binding motif ‘CC/CSSH’ is conserved within phylogenetic clades that harbor the only known thiol-specific and copper-sensitive ORs

**Fig. 8:**
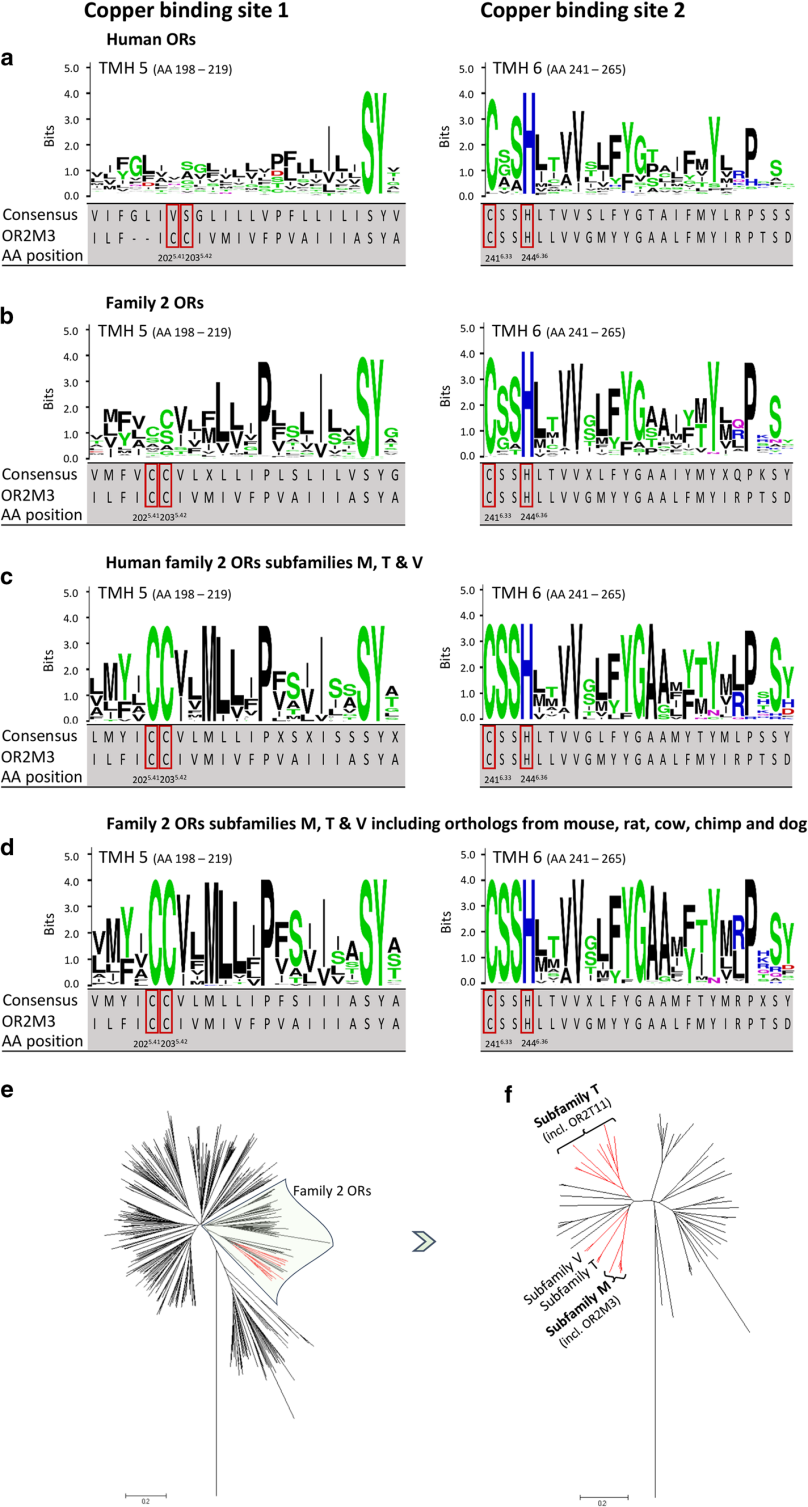
A highly conserved putative copper-binding motif in the family 2 OR subfamilies M, T, and V. Alignments of TMH 5 and TMH 6 of all human ORs (**a**), all human family 2 ORs (**b**), human family 2/subfamilies M, T, and V ORs (**c**), and family 2/subfamilies M, T, and V ORs including 99 orthologs (Tab. S5) from mouse, rat, cow, chimp, and dog (**d**). Shown are sequence logos, the consensus sequence, and the human OR2M3 sequence with the 3-mercapto-2-methylpentan-1-ol–copper-binding pocket (red boxes). The consensus amino acid refers to the most frequent one, which is determined by letter height and stacking order. The letters of each stack are ordered from the most frequent to the least frequent. Amino acid conservation is measured in bits, and 100% conservation correlates with 4.32 bits [76]. Basic amino acids (K, R, H) are blue, polar (G, S, T, Y, C) are green, hydrophilic (Q, N) are purple, acidic (D, E) are red, and hydrophobic (A, V, L, I, P, W, M, F) are black. (**e**) Phylogenetic tree of all human ORs. Family 2 ORs are shaded in green. Clades marked in red carry both sites of the conserved motif. (**f**) Phylogenetic sub-tree of family 2 ORs. Clades marked in red carry both sites of the conserved Cu^2+^-binding motif

The identified putative copper-binding motif in OR2M3, comprising site 1 (Cys202^5.41^/Cys203^5.42^) and site 2 (Cys241^6.33^/Ser242^6.34^/Ser243^6.35^/His244^6.36^), is 100% conserved among its closest homologs in 42 mammalian species (Fig. S11), and among 23 receptors within 3 subfamilies of family 2 human ORs, namely subfamilies M, T, and V (Fig. 8c). The closest 60 homologs of the 23 subfamily M/T/V ORs across 5 species (Table S5) show 100% conservancy in the CC/CSSH motif, with one exception: the dog ortholog of OR2T11 has CC/CFSH (Fig. 8d). Indeed, subfamily T of family 2 ORs harbors the only other known thiol-specific receptor, OR2T11—its activation by 2-methyl-2-propanethiol depended on the presence of copper [31] (Fig. 8e, f). Position Thr105^3.33^ (TMH 3), that supposedly forms one H-bond with the ligand in OR2M3, is conserved in only 33% among 42 orthologs, but not at all conserved in paralogs of OR2M3 (Fig. S11). Cys202^5.41^ (TMH 5), however, which forms the other H-bond with the ligand, is conserved in 100% of all homologs (Fig. S11).

In contrast, broadly tuned OR2W1 does not depend on copper for responding to its thiol ligands, and lacks site 1 (‘CC’) of the putative copper-binding motif (see Fig. 2b, d, d, f). Here, both cysteines are replaced by Leu202^5.41^/Gly203^5.42^, which lack a sulfhydryl group, and, therefore, are unlikely to interact with copper (Fig. S12). Moreover, site 2 (‘CSSH’) in OR2W1 lacks the first serine (Cys241^6.33^/Gly242^6.34^/Ser243^6.35^/His244^6.36^).

## Discussion

Thiols are important carriers of information–as key food odorants, determining the aroma of foods [6, 21], as body odors [18, 19], or as environmental odors [20]. The observation that thiols, compared to other volatiles, frequently display particularly low odor thresholds [23–25, 101], sprouted various theories trying to explain this behavior, with the aim to gain an understanding of odorant information coding at the receptor level. Common to all of these theories is the association of a thiol–receptor interaction with participation of transition metal ions such as copper, zinc, or nickel [27–31, 46, 47]. However, so far, only a single human cognate thiol odorant/receptor pair was identified (2-methyl-2-propanethiol/OR2T11, [31]), which could be used to put these theories to the test.

In the present study, we now identified OR2M3 as a further copper-sensitive human receptor, which showed a three-to-sixfold potentiation of its specific ligand’s efficacy by copper and silver ions (and colloidal silver), although this cognate receptor/ligand combination also functions in the absence of supplemented metal ions, as shown previously [6].

In an aqueous solution, Cu^2+^ is more stable than Cu^+^ (in spite of Cu^+^ having a filled d-subshell), because the solvation energy of Cu^2+^ is significantly larger than the solvation energy of Cu^+^ and thus overcompensates the second ionization energy. For silver, however, the relative energies of the two oxidation states are switched, namely, Ag^+^ is more stable than Ag^2+^. The reason for this is that the filled 4d shell of Ag^+^ is not sufficiently effective at shielding the nuclear charge making the second ionization energy so high that is not compensated by the solvation energy of Ag^2+^. As a result, silver is usually found as Ag^+^ in aqueous environments, forming rather unstable complexes with very low coordination numbers (e.g., 2). However, the incomplete solvation of Cu^2+^ in the constrained cavity of the ligand-binding site of an OR might make Cu^+^ the more stable form with a filled d-subshell. Therefore, it is natural to expect that silver could mimic copper as found by activation of OR2T11, which effect was also modeled computationally [31]. In addition, it is well known that both silver [102] and copper clusters bind thiolates [103], so they are expected to produce similar effects in the OR cavities that are sufficiently large as to bind metallic nanoparticles. In part, the significant effect of silver is due to the fact that, unlike copper, there is no background silver in the cell culture medium, so the effect of added silver appears to be larger. Because nanoparticulate silver is an environmental contaminant, our findings on the interaction of silver NP with ORs may be relevant to deleterious exposure of aquatic animals/fish/birds to environmental silver.

We recently identified OR2M3 as a highly specific, narrowly tuned receptor for the thiol KFO 3-mercapto-2-methylpentan-1-ol [6]. In contrast, for the other thiol-specific human receptor, OR2T11, its activation by 2-methyl-2-propanethiol has been previously identified to entirely depend on the presence of copper ions [31]. Li et al. [31] showed that OR2T11 responded to nine monothiols or α-mercaptothioethers, although this receptor has not been characterized to be narrowly or broadly tuned with respect to its natural ligand spectrum [31]. Thiols, however, have been shown to also activate broadly tuned receptors, i.e., OR1A1 and OR2W1 [5]. Notably, in the present study, we did not observe any potentiating effect of copper on their thiol agonists 2-phenylethanethiol and 3-mercaptohexyl acetate. In the case of 2–phenylethanethiol activating OR1A1, the presence of copper ions rather decreased its efficacy, suggesting a negative allosteric action of copper on this cognate ligand/receptor combination. In contrast, copper ions markedly reduced the potency of the same odorant in activating OR2W1, here suggesting an orthosteric competitive action of copper ions on a 2-phenylethanethiol/receptor interaction. The lack of any enhancing effect of copper on the efficacy or affinity of a homologous series of C_5_-C_8_ aliphatic thiols on OR2W1 has recently been shown by Li et al. [31].

Our results support the notion that narrowly tuned receptors with a specificity for certain metal-coordinating thiols, e.g., OR2M3, have fewer degrees of freedom in accommodating multiple ligands in their binding pocket, which may be one causative factor that determines narrow tuning in these ORs. Indeed, our modeling study revealed that the accessible volume in OR2M3 for its ligand is smaller by a factor of 1.6 than the accessible volume in OR2W1 for its three investigated ligands. For non-metal-coordinating ORs, previous studies demonstrated receptor responses to depend on the molecular volume of an odorant, showing that affinity and/or efficacy became optimal when the molecular volume of an odorant matches the size of its binding pocket within the receptor [104, 105]. Baud et al. [50] reported on two mouse receptors, Olfr73 and Olfr74, to be broadly and narrowly tuned, respectively [50]. Broadly tuned Olfr73 in their hands, however, had the smaller calculated accessible volume compared to narrowly tuned Olfr74, which they attributed to smaller ligand sizes [50].

Our model of OR2M3 predicted two amino acid positions that may form H-bonds with the ligand 3-mercapto-2-methylpentan-1-ol, Thr105^3.33^, and Cys202^5.41^. Of these, Thr105^3.33^ has also ligand-binding function in human receptors OR1A1 (Ile105^3.33^) [64], OR1G1 (Met105^3.33^) [9], and OR3A1 (His108^3.33^) [106], and in the mouse receptors Olfr73 (MOR174-9; mOR-EG) and Olfr74 (MOR144-4; mOR-EV) (Cys106^3.33^) [50, 98] (Fig. S13, Table 4). A ligand-binding function of position Cys202^5.41^ was previously reported for human OR1G1 (Ile201^5.41^) and OR7D4 (Ala202^5.41^) [97] and for mouse Olfr544 (MOR42-3) (Thr205^5.41^) [107] (summarized in Fig. S13 and Table 4). Of these two ligand-interacting amino acid positions in OR2M3, Thr105^3.33^ overlaps with a modeled, generalized odorant-binding pocket in ORs, proposed by Man et al. [88]. For OR2W1, our model suggested two amino acid positions to be involved in ligand binding (summarized in Fig. S13 and Table 5), one of which (Tyr252^6.44^) overlaps with the 22 amino acid residues proposed by Man et al. [88]. Depending on the polarity of a ligand, the binding pocket in OR2W1 supports hydrophobic contacts with non-polar amino acid residues, which has been suggested as the dominant mode of interaction between ligands and broadly tuned ORs, favoring multiple binding modes through opportunistic interactions [9]. Our data may very well be interpreted in line with such a concept of multiple ligand-specific binding modes, which may induce different OR conformations and signaling responses, and thus may be the mechanistic basis for ORs to be broadly tuned [9, 50].

**Table 4:** Amino acids constituting an odorant and copper-binding site in OR2M3

TMH segment^a^	AA position within TMH segment^a^	Mutagenesis-validated 3-Mercapto-2-methyl-pentan-1-ol binding site positions	3-Mercapto-2-methyl-pentan-1-ol	Copper	3-Mercapto-2-methyl-pentan-1-ol and copper	Validated positions in other ORs
TMH 3	8	^b,c^Thr105^3.33^	H-bond	Indirect	H-bond	His105^3.33^: [30], His108^3.33^: [31], Ile105^3.33^: [64], Met105^3.33^: [9], His108^3.33^: [106], Cys106^3.33^: [50, 98]
TMH 3	21	Met118^3.46^	No interaction	Direct	Direct	Met115^3.46^: [31]
TMH 5	5	^c^Cys202^5.41^	H-bond	Direct	H-bond via copper	Asn202^5.41^: [30], Thr202^5.41^: [9], Asn202^5.41^: [97], Thr205^5.41^: [95]
TMH 5	6	^b^Cys203^5.42^	No interaction	Direct	H-bond via copper	Thr203^5.42^: [97], Val206^5.42^: [107], Phe207^5.42^: [95]
TMH 6	1	^c^Cys241^6.33^	Indirect	Direct	Direct	Cys238^6.33^: [31]
TMH 6	4	His244^6.36^	No interaction	Direct	Direct	His241^6.36^: [31]

^a^HORDE [81]

^b^Proposed amino acid positions constituting an odorant-binding site according to Man et al. [88]

^c^3-Mercapto-2-methylpentan-1-ol-binding site in OR2M3

**Table 5:** Amino acids constituting an odorant and copper-binding site in OR2W1

TMH segment^a^	AA position within TMH segment^a^	Mutagenesis-validated binding site positions	2-Phenyl-ethanethiol	3-Mercaptohexyl acetate	Allyl phenyl acetate	Validated positions in other ORs
TMH 3	8	^b^Met105^3.33^	Indirect	Indirect	Indirect	His105^3.33^ [30], His108^3.33^ [31], Ile105^3.33^: [64], Met105^3.33^: [9], His108^3.33^: [106], Cys106^3.33^: [50, 98]
TMH 3	12	^b^Ser109^3.37^	Indirect	Indirect	Indirect	Cys109^3.37^: [30], Asn109^3.37^: [10, 94], Val113^3.37^: [107, 123]
TMH 3	15	^b^Cys112^3.40^	Indirect	Indirect	Indirect	Ser112^3.40^: [10], Ser113^3.40^: [50, 98, 117], Cys116^3.40^: [123]
TMH 6	12	^b^Tyr252^6.44^	π-π staking	No interaction	No interaction	Tyr251^6.44^: [10, 94], Tyr256^6.44^: [95], Tyr252^6.44^: [96]
TMH 6	19	Tyr259^6.51^	No interaction	H-bond	H-bond	Tyr260^6.51^: [64], Tyr258^6.51^: [64], Tyr259^6.51^: [97], Tyr260^6.51^: [50, 98], Ser263^6.51^: [95]

^a^HORDE [81]

^b^Proposed amino acid positions constituting an odorant-binding site according to Man et al. [88]

Beyond modeling the ligand-bound receptor, in silico docking of heavy metal ions into an OR model is a further challenge. The amino acid cysteine has a thiol function, and many cofactors in proteins and enzymes feature cysteinate-metal cofactors. A cysteine residue in ECL 2, Cys179, plays a major role in the tertiary structure of ORs by forming a disulfide bond with TMH 3 [86–88]. The same cysteine has been suggested to play a central role in the hypothesized metal-coordinating HxxC[DE] motif of ECL 2 [28]. In our hands, Cys179 mutants showed a complete loss-of-function, in the absence or presence of copper ions. Moreover, our docking-model did not suggest a direct interaction of Cys179 with the ligand or the copper ion, consonant with the idea of Cys179 rather being a structural requirement for ORs.

In the present study, our model of OR2M3, together with site-directed mutagenesis and functional testing of OR2M3 mutants, instead identified two positions, Cys202^5.41^ and Cys203^5.42^, constituting copper-coordinating binding site 1 (‘CC’). Another two positions, Cys241^6.33^ and His244^6.36^, supposedly constitute copper-coordinating binding site 2 (‘CSSH’). The latter one has been identified as a copper-coordinating site also in OR2T11 (‘CSSHL’) [31]. In the same study, another copper-coordinating site has been proposed in the cytoplasmic regions of TMH2 and TMH4 of OR2T11 [31]. In our study, the presence of two copper-binding sites in OR2M3 is corroborated by steeper concentration–response curves of its agonist 3-mercapto-2-methylpentan-1-ol in the presence of copper, with a Hill coefficient of 1.9, suggesting their cooperativity [108]. The observation that copper concentration-dependently inhibited its potentiation of a 3-mercapto-2-methylpentan-1-ol-dependent activation of OR2M3 may be due to the ability of copper to coordinate the thiol, removing it and making it unavailable to activate the receptor. Copper-coordinating site 2 (‘CSSH’) in our model overlaps with the ‘ionic lock’ region at the cytoplasmic end of TMH6 of GPCRs, involved in G protein interaction (for review, see [109, 110]). Also in ORs, this motif has been suggested as a zinc-binding motif, involved in G protein interaction [34]. GPCRs are allosterically modulated receptors [111, 112], often displaying constitutive activity [113, 114]. For the first time, here, we show that copper concentration-dependently inhibited a constitutive activity of OR2M3 in the absence of ligand. This suggests that copper acts as an inverse agonist on OR2M3. To validate an inverse agonist action of copper on ORs, further studies, using the [^35^S]GTP-gamma-S binding assay [115], may reveal the effect of copper on ORs’ constitutive activation of their heterotrimeric G protein. Our results also support the notion of an allosteric, ligand-independent interaction of copper with site 2 (‘CSSH’) in this receptor. Our findings are in line with reports on copper and other transition metals as dynamic allosteric regulators of protein function at external allosteric sites [42].

Knowledge of the protein structure of a receptor is critical for an understanding of its ligand interactions. However, no high-resolution crystal structure of an OR has so far been reported. Given that ORs have only about 25% sequence identity with class A GPCRs, homology modeling of ORs may be of limited informative value. Nevertheless, the strategy of combining site-directed mutagenesis, functional experimental analysis, in silico homology modeling, and docking simulations has proven successful in uncovering mechanisms of odorant/receptor interactions and OR structure–function relationships [10, 30, 31, 94, 96, 98, 107, 116–118]. A phylogenetic analysis will, therefore, add information on the relevance of conserved amino acid positions or motifs in ORs [96, 119–122].

In the present study, our phylogenetic analysis demonstrates that at least the cysteine and histidine of site 2 of the putative copper-binding motif (‘CxxH’) are conserved in all ORs. The entire ‘CSSH’ motif, however, is highly conserved in human family 2 ORs, and 100% conserved only within subfamilies ‘M, T, V’ of family 2 ORs, which harbor the closest human homologs of OR2M3 [6], and of OR2T11, the only other copper-sensitive human receptor reported, so far. In contrast, both cysteines of site 1 are not conserved over family 2 ORs or all human ORs. However, both sites together are 100% conserved only in human receptors of subfamilies ‘M, T, V’ of family 2 ORs, but also in their orthologs from, e.g., chimp, mouse, or cow. Our phylogenetic and mutational analysis, and our homology modeling/docking studies, altogether suggests that the entire motif (‘CC’/‘CSSH’) is necessary for a potentiating effect of copper, and predicts members from at least these three subfamilies of human ORs to be narrowly tuned, thiol-specific, and copper-modulated receptors. Further experiments are needed to identify the ligands for at least all family 2 ORs, and to clarify whether a copper-sensitive, specific detection of thiol odorants is idiosyncratic to human subfamilies M, T, and V of family 2 ORs, and their orthologs.

Recently, however, an enhancing effect of copper on the odorant activation of mouse receptors Olfr1509, Olfr1508, and Olfr1019 has been demonstrated [30–32], albeit these receptors lack site 1 (in our model: Cys202/Cys203 in TMH5, ‘CC’) and possess only a ‘CxxH’ site 2. Here, other QM/MM- and site-directed mutagenesis-based copper-coordinating amino acids have been proposed. The corresponding human orthologs are from families 4 and 5 of ORs, suggesting that different copper-binding sites within ORs may have developed in different phylogenetic clades.

Here, we show that the specific thiol function of human OR2M3 is modulated by copper ions. Our homology modeling/docking studies together with receptor functional expression studies suggest that this copper sensitivity is mediated by two copper-binding sites within narrowly tuned OR2M3. This putative copper-binding motif is exclusively found in subfamilies M, T, and V of family 2 ORs, and appears to be conserved across their mammalian orthologs, suggesting a conserved copper-sensitive and specific thiol function of these receptors.

### Electronic supplementary material

Below is the link to the electronic supplementary material.

Supplementary material 1 (DOCX 8647 kb)

## Abbreviations

AA: Amino acid
ECL: Extracellular loop
GPCR: G-protein coupled receptor
KFO: Key food odorant
MAF: Minor allele frequency
MC: Melanocortin receptor
OR: Odorant receptor
OSN: Olfactory sensory neuron
SNP: Single-nucleotide polymorphism
TMH: Transmembrane helix
