# Supplementary material for: Copper-mediated thiol potentiation and mutagenesis-guided modeling suggest a highly conserved copper-binding motif in human OR2M3
Source: Cell Mol Life Sci. 2019 Aug 21;77(11):2157–79. doi: 10.1007/s00018-019-03279-y (PMC7256108; doi:10.1007/s00018-019-03279-y)
Supplement: Supplementary file 1 — Supplementary material 1 (DOCX 8647 kb) [file 18_2019_3279_MOESM1_ESM.docx]

**Copper-mediated thiol potentiation and mutagenesis-guided modeling suggest a highly conserved copper binding motif in human OR2M3**

Franziska Haag^1^, Lucky Ahmed^2^, Krystle Reiss^2^, Eric Block^3^, Victor S. Batista^2^ and Dietmar Krautwurst^1^

^1^Leibniz-Institute for Food Systems Biology at the Technical University of Munich, Lise-Meitner-Str. 34, D-85354 Freising, Germany

^2^Department of Chemistry, Yale University, New Haven, CT 06520, United States

^3^Department of Chemistry, University at Albany, State University of New York, Albany, NY 12222, United States

**Contents:**

[Table S1: Oligonucleotides for molecular cloning of odorant receptors investigated. 2](#_Toc10700365)

[Table S2: Oligonucleotides for *Homo sapiens* OR2M3 site-directed mutagenesis. 2](#_Toc10700366)

[Table S3: Oligonucleotides for *Homo sapiens* OR2W1 site-directed mutagenesis. 4](#_Toc10700367)

[Table S4: Vector internal oligonucleotides for pi2-dk (39aa rho-tag). 4](#_Toc10700368)

[Table S5: NCBI reference sequences of olfactory receptor genes investigated. 5](#_Toc10700369)

[Table S6: EC_50_ values and relative amplitudes for 3-mercapto-2-methylpentan-1-ol on OR2M3 wild type in the absence and presence of different heavy metals. 7](#_Toc10700370)

[Table S7: EC_50_ values and relative amplitudes for OR2M3 wild type in the absence and presence of different concentrations of Cu^2+^. 7](#_Toc10700371)

[Fig. S1. OR2M3 modeled 3-dimensional structure and its template. 8](#_Toc15993904)

[Fig. S2. TMHMM posterior probabilities for OR2M3. 8](#_Toc15993905)

[Fig. S3. OR2W1 and OR2M3 modeled 3-dimensional structures and their template. 9](#_Toc15993906)

[Fig. S4. TMHMM posterior probabilities for OR2W1. 9](#_Toc15993907)

[Fig. S5. Cα-RMSD plot of OR2W1. 10](#_Toc15993908)

[Fig. S6. Copper and silver enhance the 3-mercapto-2-methylpentan-1-ol efficacy on OR2M3. 11](#_Toc15993909)

[Fig. S7. The strongest increase in efficacy of 3-mercapto-2-methylpentan-1-ol on OR2M3 was observed with 10 μmol/L Cu^2+^. 12](#_Toc15993910)

[Fig. S8. Cu^2+^ influences the efficacy of 3-mercapto-2-methylpentan-1-ol on OR2M3 but not of OR2W1 agonists. 13](#_Toc15993911)

[Fig. S9. SNP-defined OR2M3 haplotypes display 3-mercapto-2-methylpentan-1-ol gain- and loss-of-function phenotypes. 14](#_Toc15993912)

[Fig S10. Molecular models of the ligand and copper binding cavity for OR2M3. 15](#_Toc15993913)

[Fig. S11. A conserved copper binding motif within OR2M3 homologs. 16](#_Toc15993914)

[Fig. S12. Copper binding site within different ORs. 17](#_Toc15993915)

[Fig. S13. Odorant binding site within different ORs. 18](#_Toc15993916)

[Fig. S14: Cell surface expression of OR2M3 wt and variants. 19](#_Toc15993917)

Table S1: Oligonucleotides for molecular cloning of odorant receptors investigated.

| **Gene** | **Oligo-nucleotide** | **Restriction Site** | **TM (°C)** |  | **Sequence 5´🡪3´** |
| --- | --- | --- | --- | --- | --- |
| hsOR1A1 | 344 | MfeI | 66 | fw | CGAT *CAATTG* **ATG** AGG GAA AAT AAC CAG TCC TCT ACA CTG GAA TTC ATC C |
|  | cg-196 | NotI | 61 | rv | CTGC *GCGGCCGC* **TTA** CGA GGA GAT TCT CTT GTT GAA GAG TTT CC |
| hsOR2M3 | jf-071 | MfeI | 64 | fw | GTCG *CAATTG* **ATG** GCA AGG GAG AAT TCG ACC TTC AAC TCC G |
|  | jf-072 | NotI | 63 | rv | CTGC *GCGGCCGC* **TCA** CTC TCC AGA CTT GCC CTT TCC TAA GAT C |
| hsOR2W1 | dk-807 | EcoRI | 58 | fw | GTGA *GAATTC* **ATG** GAC CAA AGC AAT TAT AGT TCT TTA CAT GG |
|  | dk-808 | NotI | 59 | rv | GCTAA *GCGGCCGC* **CTA** TGA CTT GCA ATT CCT CTT TAT TTT TGT AGA TTT G |

TM = melting temperature, fw = forward, rv = reverse. Italic letters highlight the restriction sites. Start and Stop codons are bold.

Table S2: Oligonucleotides for *Homo sapiens* OR2M3 site-directed mutagenesis.

| **Gene** | **Oligonucleotide** | **TM (°C)** |  | **Sequence 5´🡪3´** |
| --- | --- | --- | --- | --- |
| OR2M3 Y_104_C | FN-101 | 59 | fw | *CAC AAA TTT TCT TCT GTA CAT CAC TGC TTG G* |
|  | FN-102 | 60 | rv | *GCA GTG ATG TAC AGA AGA AAA TTT GTG TGG C* |
| OR2M3 T_105_A | FN-105 | 61 | fw | *CAC AAA TTT TCT TCT ATG CAT CAC TGC TTG GC* |
|  | FN-106 | 60 | rv | *GCA GTG ATG CAT AGA AGA AAA TTT GTG TGG C* |
| OR2M3 T_105_H | FN-051 | 59 | fw | *CAA ATT TTC TTC TAT CAC TCA CTG CTT GGC* |
|  | FN-052 | 60 | rv | *CAG AGC CAA GCA GTG AGT GAT AGA AG* |
| OR2M3 T_105_I | FN-103 | 60 | fw | *CAA ATT TTC TTC TAT ATA TCA CTG CTT GGC TCT G* |
|  | FN-104 | 60 | rv | *GCC AAG CAG TGA TAT ATA GAA GAA AAT TTG TGT G* |
| OR2M3 T_105_V | FN-191 | 59 | fw | *CAC AAA TTT TCT TCT ATG TAT CAC TGC TTG GC* |
|  | FN-192 | 58 | rv | *CAA GCA GTG ATA CAT AGA AGA AAA TTT GTG TG* |
| OR2M3 G_109_C | FN-053 | 58 | fw | *CAT CAC TGC TTT GCT CTG AAT GCT TTC* |
|  | FN-054 | 59 | rv | *CAA AAG AAA GCA TTC AGA GCA AAG CAG TG* |
| OR2M3 G_109_R | FN-131 | 61 | fw | *CTA TAC ATC ACT GCT TCG CTC TGA GTG C* |
|  | FN-132 | 60 | rv | *GAA AGC ACT CAG AGC GAA GCA GTG* |
| OR2M3 C_112_A | FN-047 | 59 | fw | *GCT TGG CTC TGA GGC CTT TCT TTT G* |
|  | FN-048 | 59 | rv | *GCC AAA AGA AAG GCC TCA GAG CC* |
| OR2M3 C_112_S | FN-049 | 58 | fw | *GCT TGG CTC TGA GAG CTT TCT TTT G* |
|  | FN-050 | 57 | rv | *GCC AAA AGA AAG CTC TCA GAG CC* |
| OR2M3 M_118_A | FN-235 | 61 | fw | *CTT TTG GCT GTT GCG GCT TAT GAC CG* |
|  | FN-236 | 64 | rv | *GTA GCG GTC ATA AGC CGC AAC AGC CAA AAG* |
| OR2M3 D_159_V | FN-133 | 59 | fw | *CTA CGG ATG GAA TTA TTG TTG TTG TAG CAA C* |
|  | FN-134 | 60 | rv | *CTA CAA CAA CAA TAA TTC CAT CCG TAG AGC C* |
| OR2M3 C_179_Y | FN-149 | 61 | fw | *CCC ACT TCT TCT ATG ACT TCC CCT CC* |
|  | FN-150 | 61 | rv | *GTA GGG AGG GGA AGT CAT AGA AGA AGT G* |
| OR2M3 D_180_E | FN-151 | 61 | fw | *CCA CTT CTT CTG TGA ATT CCC CTC CC* |
|  | FN-152 | 60 | rv | *GTA GGG AGG GGA ATT CAC AGA AGA AG* |
| OR2M3 D_180_N | FN-171 | 61 | fw | *CCA CTT CTT CTG TAA CTT CCC CTC CC* |
|  | FN-172 | 61 | rv | *GAG GGG AAG TTA CAG AAG AAG TGG GC* |

| **Table S2 continued** | |  |  |  |
| --- | --- | --- | --- | --- |
| OR2M3 C_202_S | FN-039 | 59 | fw | *GAA AAG ATT CTT TTC ATC AGC TGT ATA GTA ATG ATT G* |
|  | FN-040 | 59 | rv | *GAA AAC AAT CAT TAC TAT ACA GCT GAT GAA AAG AAT C* |
| OR2M3 C_202_A | FN-041 | 57 | fw | *GAT TCT TTT CAT CGC ATG TAT AGT AAT GAT TG* |
|  | FN-042 | 57 | rv | *GAA AAC AAT CAT TAC TAT ACA TGC GAT GAA AAG* |
| OR2M3 C_203_A | FN-045 | 58 | fw | *CTT TTC ATC TGC GCA ATA GTA ATG ATT GTT TTC* |
|  | FN-046 | 59 | rv | *GGG AAA ACA ATC ATT ACT ATT GCG CAG ATG* |
| OR2M3 C_203_S | FN-043 | 57 | fw | *GAT TCT TTT CAT CTG CAG TAT AGT AAT GAT TG* |
|  | FN-044 | 57 | rv | *GAA AAC AAT CAT TAC TAT ACT GCA GAT GAA AAG* |
| OR2M3 C_203_Y | FN-107 | 58 | fw | *GAA AAG ATT CTT TTC ATC TGC TAT ATA GTA ATG ATT G* |
|  | FN-108 | 59 | rv | *GGA AAA CAA TCA TTA CTA TAT AGC AGA TGA AAA GAA TC* |
| OR2M3 M_206_A | FN-241 | 58 | fw | *CTG CTG TAT AGT AGC GAT TGT TTT CCC* |
|  | FN-242 | 58 | rv | *CAG GGA AAA CAA TCG CTA CTA TAC AGC* |
| OR2M3 M_206_I | FN-109 | 59 | fw | *CAT CTG CTG TAT AGT AAT CAT TGT TTT CCC TG* |
|  | FN-110 | 61 | rv | *GCA ACA GGG AAA ACA ATG ATT ACT ATA CAG CAG* |
| OR2M3 I_207_L | FN-111 | 60 | fw | *CTG CTG TAT AGT AAT GCT GGT TTT CCC TG* |
|  | FN-112 | 60 | rv | *GCA ACA GGG AAA ACC AGC ATT ACT ATA CAG* |
| OR2M3 C_241_A | FN-237 | 60 | fw | *GCT TTT ACT ACT GCT TCC TCT CAC CTC* |
|  | FN-238 | 60 | rv | *GTG AGA GGA AGC AGT AGT AAA AGC TTT GC* |
| OR2M3 H_244_A | FN-239 | 61 | fw | *CTG CTT CCT CTG CCC TCT TGG TG* |
|  | FN-240 | 63 | rv | *CAC CAC CAA GAG GGC AGA GGA AGC* |
| OR2M3 A_255_G | FN-135 | 61 | fw | *GTA CTA TGG AGC AGG TTT GTT CAT GTA CAT AC* |
|  | FN-136 | 61 | rv | *GCC GTA TGT ACA TGA ACA AAC CTG CTC C* |
| OR2M3 M_258_T | FN-185 | 59 | fw | *GCA GCT TTG TTC ACG TAC ATA CGG C* |
|  | FN-186 | 61 | rv | *GTG GGC CGT ATG TAC GTG AAC AAA GC* |
| OR2M3 Y_278_C | FN-197 | 60 | fw | *GTG TCT GTA TTC TGC ACC ATC CTC AC* |
|  | FN-198 | 61 | rv | *GAG GAT GGT GCA GAA TAC AGA CAC CAT C* |
| OR2M3 P_287_S | FN-187 | 62 | fw | *CTC CCA TGT TGA ATT CCC TCA TCT ACA GC* |
|  | FN-188 | 60 | rv | *GAG GCT GTA GAT GAG GGA ATT CAA CAT G* |

TM = melting temperature, fw = forward, rv = reverse.

Table S3: Oligonucleotides for *Homo sapiens* OR2W1 site-directed mutagenesis.

| **Gen** | **Oligonucleotide** | **TM (°C)** |  | **Sequence 5´🡪3´** |
| --- | --- | --- | --- | --- |
| OR2W1 M_105_H | FN-205 | 60 | fw | *CTC TAT GTT TAC CAC TGG TTG GGC TC* |
|  | FN-206 | 60 | rv | *CAA CTC TAT GTT TAC CAC TGG TTG GGC* |
| OR2W1 S_109_A | FN-253 | 60 | fw | *GTG GTT GGG CGC AGT TGA GTG C* |
|  | FN-254 | 61 | rv | *GAA GGC ACT CAA CTG CGC CCA AC* |
| OR2W1 S_109_C | FN-203 | 60 | fw | *GTG GTT GGG CTG CGT TGA GTG C* |
|  | FN-204 | 59 | rv | *CTC AAC GCA GCC CAA CCA CAT G* |
| OR2W1 C_112_A | FN-201a | 61 | fw | *GCT CAG TTG AGG CCC TTC TCC TG* |
|  | FN-202a | 60 | rv | *GCC AGG AGA AGG GCC TCA ACT G* |
| OR2W1 C_179_A | FN-207 | 60 | fw | *GAT CAT TTC TTG GCT GAG TTG CCA GC* |
|  | FN-208 | 61 | rv | *CAG AGC TGG CAA CTC AGC CAA GAA ATG* |
| OR2W1 L_202_C | FN-209 | 59 | fw | *CTG TTT TCG CTT GCG GCA TTA TAA TTG TC* |
|  | FN-210 | 60 | rv | *GAC AAT TAT AAT GCC GCA AGC GAA AAC AGA C* |
| OR2W1 L_202_S | FN-211 | 59 | fw | *CTG TTT TCG CTT CCG GCA TTA TAA TTG TC* |
|  | FN-212 | 60 | rv | *GAC AAT TAT AAT GCC GGA AGC GAA AAC AGA C* |
| OR2W1 L_202_C/G_203_C | FN-243 | 61 | fw | *GTC TGT TTT CGC TTG CTG CAT TAT AAT TGT CC* |
|  | FN-244a | 61 | rv | *GTG AGG ACA ATT ATA ATG CAG CAA GCG AAA AC* |
| OR2W1 C_241_A | FN-245 | 57 | fw | *GCA ATG AAT ACC GCG GGA TCT C* |
|  | FN-246 | 58 | rv | *GAT CCC GCG GTA TTC ATT GCT TTT C* |
| OR2W1 H_244_A | FN-247 | 58 | fw | *CTG TGG ATC TGC TCT TAC TGT AGT G* |
|  | FN-248 | 58 | rv | *GAC ACT ACA GTA AGA GCA GAT CCA C* |
| OR2W1 Y_252_A | FN-249 | 59 | fw | *GTC TAT GTT CGC CGG AAC TAT TAT CTA C* |
|  | FN-250 | 58 | rv | *GAT AAT AGT TCC GGC GAA CAT AGA CAC* |
| OR2W1 Y_259_A | FN-251 | 58 | fw | *CTA CAT GGC CCT GCA ACC AGG* |
|  | FN-252 | 61 | rv | *GTT ACC TGG TTG CAG GGC CAT GTA G* |
| OR2W1 D_296_N | FN-199 | 59 | fw | *CAC CTT AAG AAA TAA GAA CAT GAA GGA TGC C* |
|  | FN-200 | 59 | rv | *CAG GGC ATC CTT CAT GTT CTT ATT TCT TAA G* |

TM = melting temperature, fw = forward, rv = reverse.

Table S4: Vector internal oligonucleotides for pi2-dk (39aa rho-tag).

| **Oligonucleotide** | **TM (°C)** |  | **Sequence 5´🡪3´** |
| --- | --- | --- | --- |
| dk-231 | 57 °C | fw | GCA GAG CTG GTT TAG TGA ACC G |
| dk-232a | 59 °C | rv | GCA AGT AAA ACC TCT ACA AAT GTG GTA TGG |

TM = melting temperature, fw = forward, rv = reverse

Table S5: NCBI reference sequences of olfactory receptor genes investigated.

| **Gene Description** | **Species** | **Common Species Name** | **NCBI Reference Sequence**  **(Accession-number)** |
| --- | --- | --- | --- |
| OR2M3 | *Ailuropoda melanoleuca* | Giant panda | XM_002930259.1 |
| OR2M3-like | *Aotus nancymaae* | Nancy Ma's night monkey | XM_012472652.1 |
| OR2M3-like | *Bison bison bison* | Plains bison | XM_010861634.1 |
| OR2M3-like | *Bos mutus* | Wild yak | XM_014480762.1 |
| OR2M3 | *Bos taurus* | Cattle | XM_015472178.1 |
| OR2M3-like | *Canis lupus familiaris* | Domestic dog | XM_014118893.1 |
| OR2M3-like | *Capra hircus* | Domestic goat | XM_013965440.2 |
| OR2M3-like | *Cavia porcellus* | Guinea pig | XM_003479747.1 |
| OR2M3 | *Ceratotherium simum simum* | Southern white rhinoceros | XM_014797638.1 |
| OR2M3 | *Cercocebus atys* | Sooty mangabey | XM_012045212.1 |
| OR2M3-like | *Chlorocebus sabaeus* | Green monkey | XM_007990109.1 |
| OR2M3-like | *Chrysochloris asiatica* | Cape golden mole | XM_006877082.1 |
| OR2M3-like | *Colobus angolensis palliatus* | Angola colobus | XM_011955784.1 |
| OR2M3 | *Dipodomys ordii* | Ord's kangaroo rat | XM_013015457.1 |
| OR2M3-like | *Echinops telfairi* | Lesser hedgehog tenrec | XM_004696864.2 |
| OR2M3-like | *Equus asinus* | Asses | XM_014851596.1 |
| OR2M3-like | *Equus caballus* | Domestic horse | XM_001493883.2 |
| OR2M3-like | *Erinaceus europaeus* | European hedgehog | XM_007516422.1 |
| OR2M3 | *Felis catus* | Domestic cat | XM_011283070.2 |
| OR2M3-like | *Galeopterus variegatus* | Malayan flying lemur | XM_008590481.1 |
| OR2M3 | *Gorilla gorilla gorilla* | Western lowland gorilla | XM_004028736.2 |
| OR2M3 | *Homo sapiens* | Human | NM_001004689.1 |
| OR2M3-like | *Ictidomys tridecemlineatus* | Thirteen-lined ground squirrel | XM_005342365.1 |
| OR2M3-like | *Loxodonta africana* | African bush elephant | XM_003423106.2 |
| OR2M3 | *Macaca fascicularis* | Crab-eating macaque | XM_005541090.2 |
| OR2M3 | *Macaca mulatta* | Rhesus macaque | XM_001094325.3 |
| OR2M3 | *Macaca nemestrina* | Southern pig-tailed macaque | XM_011729605.1 |
| OR2M3 | *Mandrillus leucophaeus* | Drill | XM_011972074.1 |
| OR2M3 | *Microcebus murinus* | Gray mouse lemur | XM_012758105.1 |
| Olfr164 | *Mus musculus* | Mouse | NM_146451.1 |
| OR2M3-like | *Nannospalax galili* | Mole rats | XM_008843779.1 |
| OR2M3-like | *Orycteropus afer afer* | Aardvark | XM_007957565.1 |
| OR2M3 | *Otolemur garnettii* | Small-eared greater galago | XM_003803649.2 |
| OR2M3-like | *Ovis aries* | Domestic sheep | XM_004008749.3 |
| OR2M3 | *Pan paniscus* | Bonobo | XM_003815318.2 |
| OR2M3 | *Pan troglodytes* | Common chimpanzee | XM_001143601.5 |
| OR2M3 | *Papio anubis* | Olive baboon | XM_003893634.2 |
| OR2M3-like | *Propithecus coquereli* | Coquerel's sifaka | XM_012647451.1 |
| Olr1570 | *Rattus norvegicus* | Rat | NM_001000041.1 |
| OR2M3 | *Sus scrofa* | Wild boar | XM_013991466.1 |
| OR2M3-like | *Tarsius syrichta* | Philippine tarsier | XM_008056882.1 |
| OR2M3 | *Tupaia chinensis* | Chinese tree shrew | XM_006165337.1 |
| OR2M3 | *Ursus maritimus* | Polar bear | XM_008710276.1 |
| Olfr165 | *Mus musculus* | Mouse | NM_146466.1 |
| Olfr31 | *Mus musculus* | Mouse | NM_147027.2 |
| **Table S5 continued** | |  |  |
| Olfr331 | *Mus musculus* | Mouse | NM_001011861.3 |
| Olfr330 | *Mus musculus* | Mouse | NM_146879.2 |
| Olfr325 | *Mus musculus* | Mouse | NM_207153.2 |
| Olfr328 | *Mus musculus* | Mouse | NM_146502.2 |
| Olfr224 | *Mus musculus* | Mouse | NM_207695.1 |
| Olfr720 | *Mus musculus* | Mouse | NM_146392.1 |
| Olfr314 | *Mus musculus* | Mouse | NM_001011760.2 |
| Olfr56 | *Mus musculus* | Mouse | NM_010999.3 |
| Olfr1396 | *Mus musculus* | Mouse | NM_146337.1 |
| Olr1606 | *Rattus norvegicus* | Rat | NM_001000502.1 |
| Olr1605 | *Rattus norvegicus* | Rat | NM_001000088.1 |
| Olr1425 | *Rattus norvegicus* | Rat | NM_001000010.1 |
| Olr1424 | *Rattus norvegicus* | Rat | NM_001000779.1 |
| Olr1607 | *Rattus norvegicus* | Rat | NM_001000534.1 |
| Olr1456 | *Rattus norvegicus* | Rat | NM_001000956.1 |
| Olr1386 | *Rattus norvegicus* | Rat | NM_214834.1 |
| Olr1384 | *Rattus norvegicus* | Rat | NM_001002291.1 |
| Olr1383 | *Rattus norvegicus* | Rat | NM_214832.1 |
| Olr1385 | *Rattus norvegicus* | Rat | NM_214833.1 |
| OR2T1 | *Bos taurus* | Cattle | XM_002689121.2 |
| OR2T2 | *Bos taurus* | Cattle | XM_024995371.1 |
| OR2T6 | *Bos taurus* | Cattle | XM_002689120.2 |
| OR2T8 | *Bos taurus* | Cattle | XM_002689092.5 |
| OR2T8 | *Bos taurus* | Cattle | XM_002689090.3 |
| OR2T11 | *Bos taurus* | Cattle | XM_002689105.4 |
| OR2T12 | *Bos taurus* | Cattle | XM_005209254.3 |
| OR2T27 | *Bos taurus* | Cattle | XM_002689122.1 |
| OR2T29 | *Bos taurus* | Cattle | XM_010807207.3 |
| OR2T33 | *Bos taurus* | Cattle | XM_002689091.2 |
| OR2V1 | *Bos taurus* | Cattle | XM_002689049.2 |
| OR2V2 | *Bos taurus* | Cattle | XM_002689052.5 |
| OR2M4 | *Pan troglodytes* | Common chimpanzee | XM_016951697.2 |
| OR2M5 | *Pan troglodytes* | Common chimpanzee | XM_016951673.1 |
| OR2T1 | *Pan troglodytes* | Common chimpanzee | XM_003308851.1 |
| OR2T2 | *Pan troglodytes* | Common chimpanzee | XM_016948111.2 |
| OR2T4 | *Pan troglodytes* | Common chimpanzee | XM_001142557.2 |
| OR2T6 | *Pan troglodytes* | Common chimpanzee | XM_016951722.1 |
| OR2T11 | *Pan troglodytes* | Common chimpanzee | XM_525162.4 |
| OR2T12 | *Pan troglodytes* | Common chimpanzee | XM_001143155.4 |
| OR2T27 | *Pan troglodytes* | Common chimpanzee | XM_525157.5 |
| OR2T29 | *Pan troglodytes* | Common chimpanzee | XM_525160.6 |
| OR2V1 | *Pan troglodytes* | Common chimpanzee | XM_016953414.2 |
| OR2V2 | *Pan troglodytes* | Common chimpanzee | XM_016953415.1 |
| OR2T1 | *Canis lupus familiaris* | Domestic dog | XM_005628360.1 |
| OR2T2 | *Canis lupus familiaris* | Domestic dog | XM_539359.3 |
| OR2T6 | *Canis lupus familiaris* | Domestic dog | XM_844720.2 |
| cOR2T18 | *Canis lupus familiaris* | Domestic dog | NM_001256452.1 |
| **Table S5 continued** | |  |  |
| OR2T27 | *Canis lupus familiaris* | Domestic dog | XM_539358.3 |
| OR2T29 | *Canis lupus familiaris* | Domestic dog | XM_539362.3 |
| OR2T33 | *Canis lupus familiaris* | Domestic dog | XM_003431849.1 |
| cOR2T15 | *Canis lupus familiaris* | Domestic dog | XM_539347.3 |
| cOR2T24 | *Canis lupus familiaris* | Domestic dog | XM_539346.3 |
| cOR2T13 | *Canis lupus familiaris* | Domestic dog | XM_539348.4 |
| OR2V1 | *Canis lupus familiaris* | Domestic dog | XM_548792.3 |

Table S6: EC_50_ values and relative amplitudes for 3-mercapto-2-methylpentan-1-ol on OR2M3 wild type in the absence and presence of different heavy metals.

| **Metal** | **EC_50_ in µmol/L^a^** | **Relative amplitude^b^** |
| --- | --- | --- |
| w/o | 0.45 ± 0.19 | 1 |
| CuCl_2_ | 0.55 ± 0.01 | 2.62 ± 0.80^c^ |
| CuCl_2_ + TEPA | 1.13 ± 0.36 | 0.21 ± 0.03 |
| CuCl_2_^d^ | 0.25 ± 0.19 | 1 |
| FeCl_3_ | 1.67 ± 0.34 | 1.04 ± 0.16 |
| NiCl_2_ | 1.09 ± 0.63 | 0.50 ± 0.12 |
| CoCl_2_ | 0.73 ± 0.26 | 0.40 ± 0.11 |
| ZnSO_4_ | n.d. | n.d. |
| AgAc | 0.87 ± 0.50 | 5.72 ± 0.42 |
| AgNO_3_ | 1.49 ± 0.36 | 6.41 ± 1.17 |
| colloidal Ag | 0.76 ± 0.35 | 3.52 ± 0.69 |

^a^mean ± SD (n=3-5). ^b^Data were normalized to the OR2M3 wt signal in response to 3-mercapto-2-methylpentan-1-ol (20 µmol/L), measured in the absence of Cu^2+^. ^c^OR2M3 wt with 3-mercapto-2-methylpentan-1-ol (6 µmol/L). ^d^OR2T11 C_119_R with 2-methylpropane-2-thiol. n.d., no detectable response up to 60 µmol/L.

Table S7: EC_50_ values and relative amplitudes for OR2M3 wild type in the absence and presence of different concentrations of Cu^2+^.

| **Copper concentration** | **EC_50_ in µmol/L^a^** | **Relative amplitude^b^** |
| --- | --- | --- |
| w/o | 0.45 ± 0.19 | 1 |
| 10 µmol | 0.29 ± 0.10 | 3.47 ± 1.90 |
| 30 µmol | 0.55 ± 0.01 | 2.62 ± 0.80^c^ |
| 60 µmol | 1.47 ± 0.12 | 1.42 ± 0.64 |
| 100 µmol | 1.95 ± 0.30 | 0.73 ± 0.48 |
| 300 µmol | n.d. | n.d. |

^a^mean ± SD (n=3-5). ^b^Data were normalized to the OR2M3 wt signal in response to 3-mercapto-2-methylpentan-1-ol (20 µmol/L), measured in the absence of Cu^2+^. ^c^OR2M3 wt with 3-mercapto-2-methylpentan-1-ol (6 µmol/L). n.d., no detectable response up to 60 µmol/L.


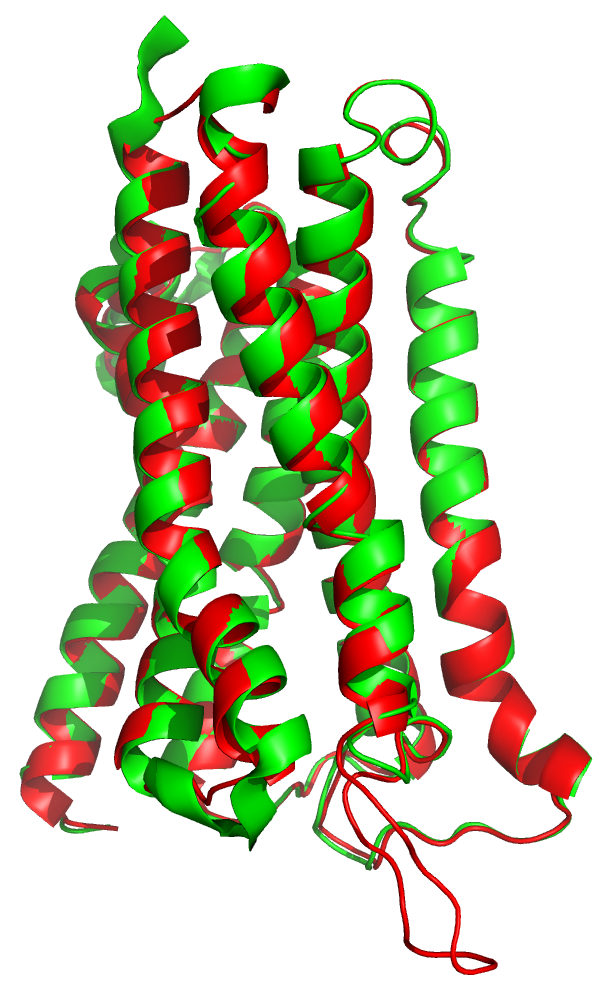


Fig. S1. OR2M3 modeled 3-dimensional structure and its template.

Sequence alignment of the seven TMH regions of the homology model of OR2M3 (red) with the human M1 muscarinic receptor (5CXV.pdb, green).


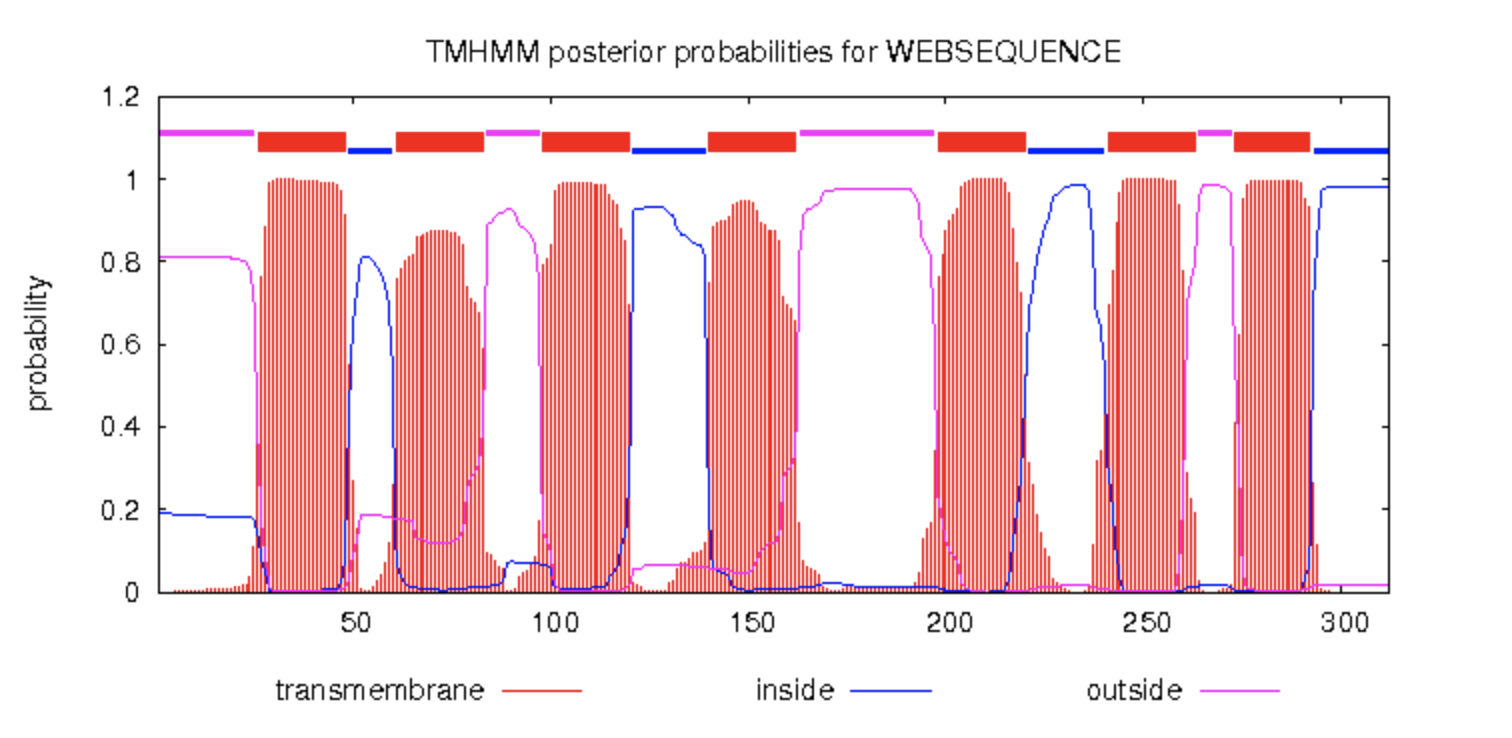


Fig. S2. TMHMM posterior probabilities for OR2M3.

The graphical output of TMHMM shows the posterior probabilities for transmembrane, inside, and outside regions. Transmembrane regions are indicated as red bars.


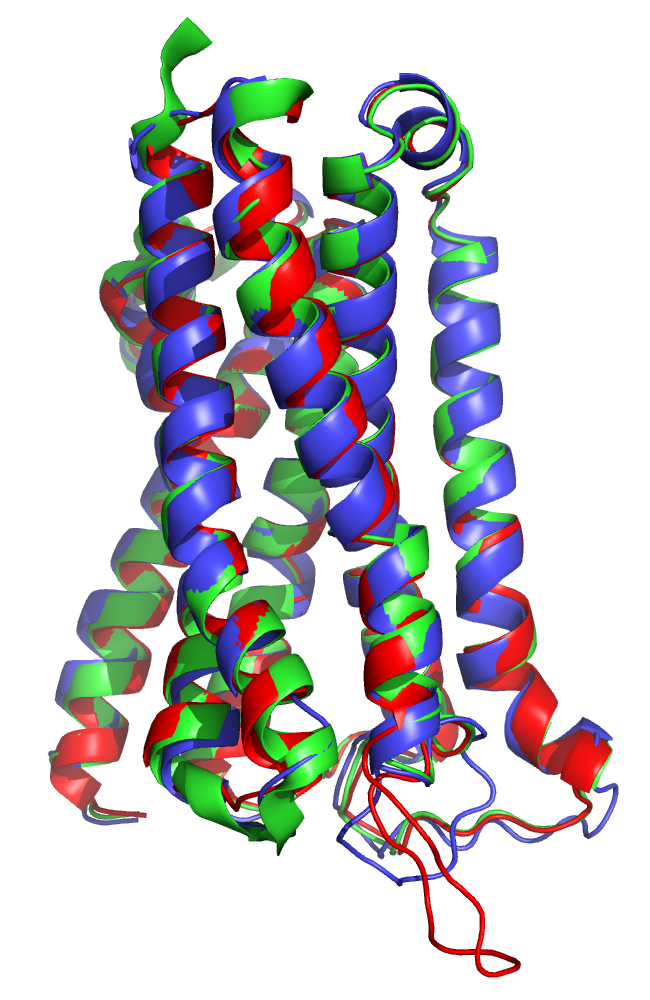


Fig. S3. OR2W1 and OR2M3 modeled 3-dimensional structures and their template.

Sequence alignment of the seven TMH regions of the homology model of OR2M3 (red) and OR2W1 (blue) with the human M1 muscarinic receptor (5CXV.pdb, green).


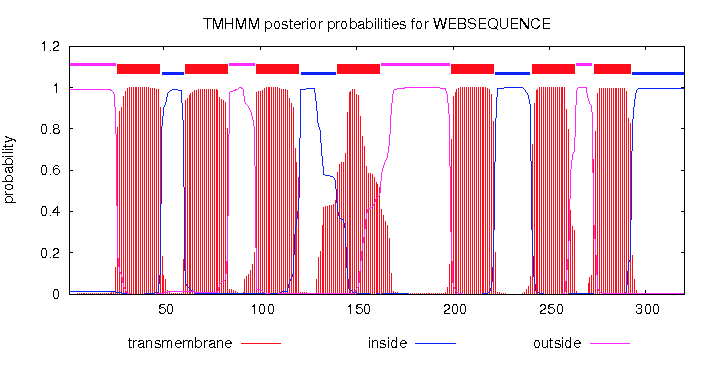


Fig. S4. TMHMM posterior probabilities for OR2W1.

The graphical output of TMHMM shows the posterior probabilities for transmembrane, inside, and outside regions. Seven transmembrane regions are indicated as red bars.


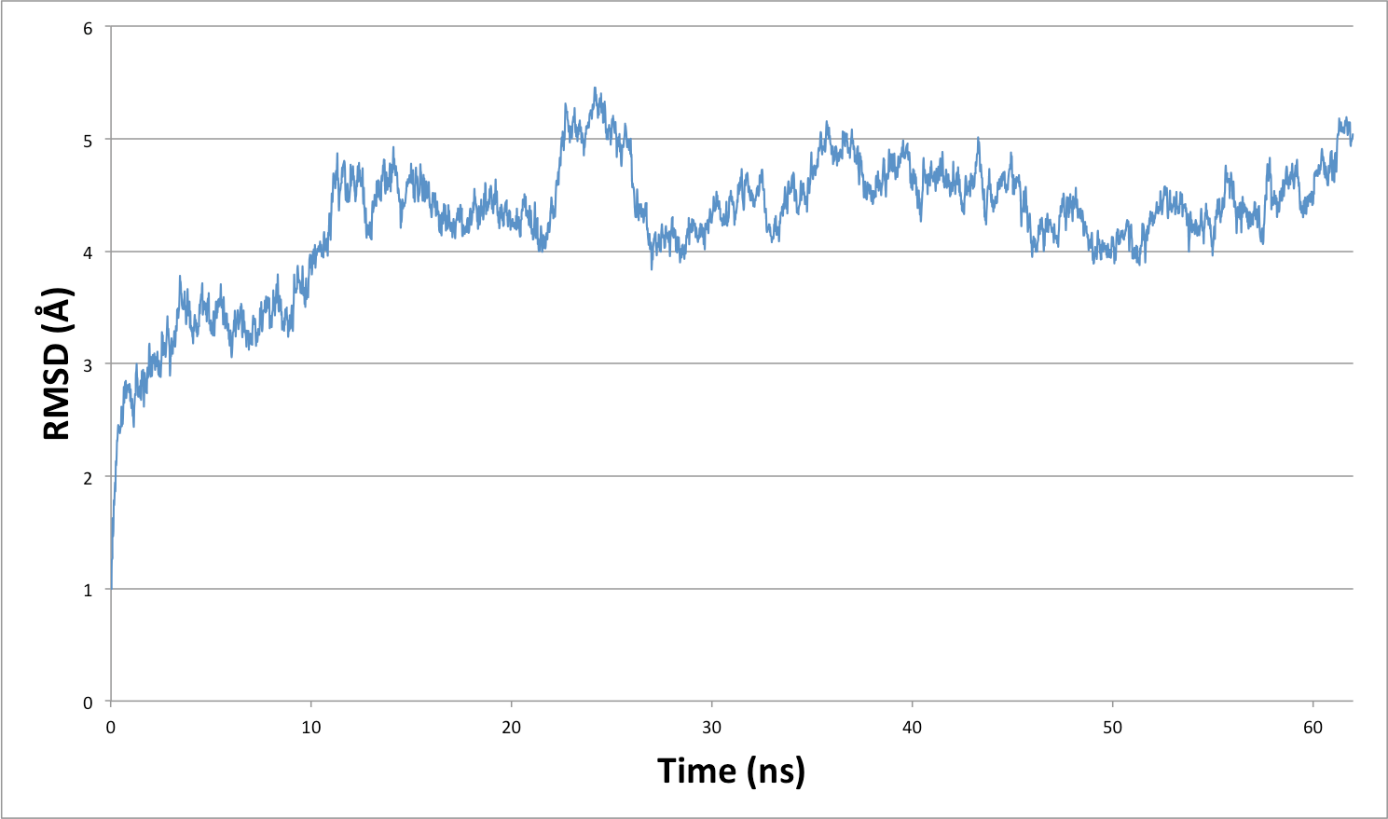


Fig. S5. C(α)-RMSD plot of OR2W1.

Shown is a 62 ns simulation of OR2W1 in a water box without a membrane, which stabilizes to ~4.5 Å after the first 12 ns.


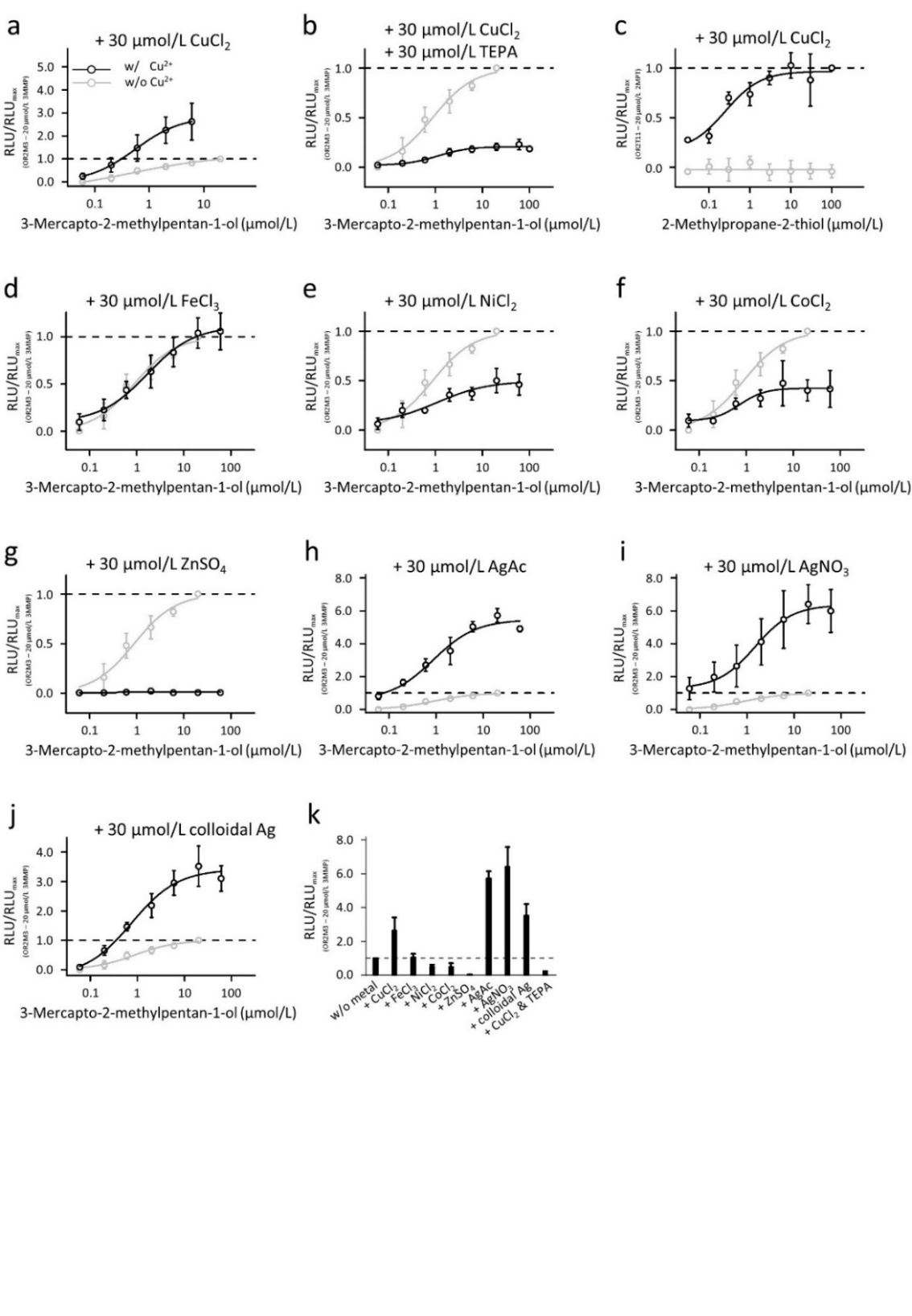


Fig. S6. Copper and silver enhance the 3-mercapto-2-methylpentan-1-ol efficacy on OR2M3.

Concentration-response relation of 3-mercapto-2-methylpentan-1-ol on OR2M3 in the absence or presence of each 30 µmol/L CuCl_2_ (a), and 30 µmol/L CuCl_2_ and the copper chelator TEPA (b). Concentration-response relation of 2-methylpropane-2-thiol on OR2T11 C_119_R in the absence or presence of 30 µmol/L CuCl_2_ (c). Concentration-response relation of 3-mercapto-2-methylpentan-1-ol on OR2M3 in the absence or presence of each 30 µmol/L FeCl_3_ (d); NiCl_2_ (e); CoCl_2_ (f); ZnSO_4_ (g); AgAc (h); AgNO_3_ (i) and colloidal silver (j). Comparison of efficacies of 20 µmol/L 3-mercapto-2-methylpentan-1-ol on OR2M3 in the absence or presence of different metal ions at 30 µmol/L (k). Note that the same data set in the absence of supplemented Cu^2+^ (grey) is given in sub-panels (a-b, d-j) for didactic reasons. Data were mock control-subtracted, normalized to the OR2M3 wt signal in response to 3-mercapto-2-methylpentan-1-ol (20 µmol/L), measured in the absence of Cu^2+^ (a-b, d-k) or rather normalized to the OR2T11 C_119_R signal in response to 2-methylpropan-2-thiol (100 µmol/L), measured in the presence of Cu^2+^ (c), and shown as mean ± SD (n = 3 – 6). RLU = relative luminescence unit. 3MMP = 3-mercapto-2-methylpentan-1-ol. Curves represent best fits to the data in the absence (grey) or presence (black) of Cu^2+^, with EC_50_ values given in Table S6. Note that for didactical reasons the concentration-response relations in Fig. 1b and Fig. S5a are the identical data set. Also Fig. 1a and Fig. S5k are the identical data set.


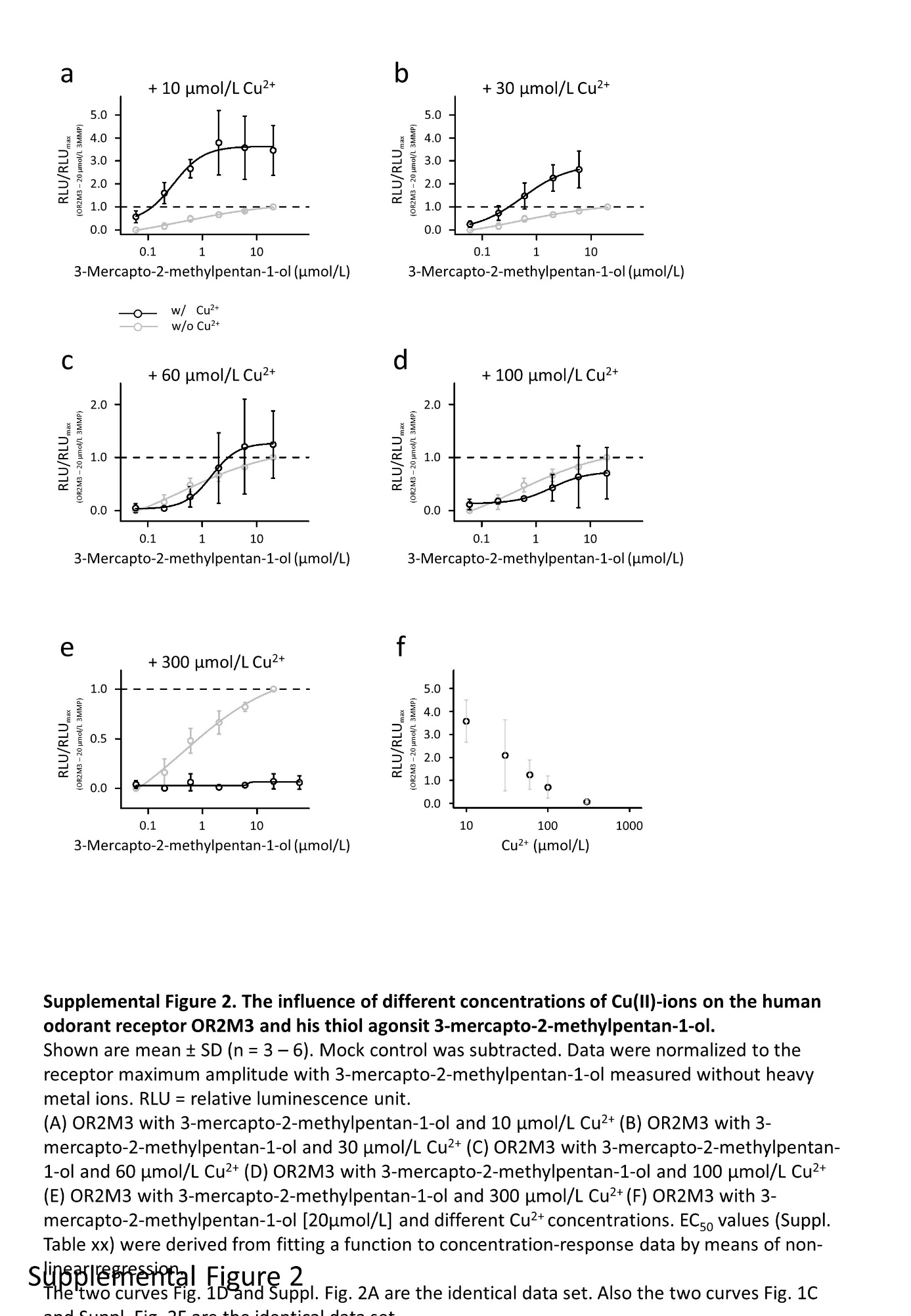


Fig. S7. The strongest increase in efficacy of 3-mercapto-2-methylpentan-1-ol on OR2M3 was observed with 10 μmol/L Cu^2+^.

Concentration-response relation of 3-mercapto-2-methylpentan-1-ol on OR2M3 in the absence or presence of 10 µmol/L CuCl_2_ (a); 30 µmol/L CuCl_2_ (b); 60 µmol/L CuCl_2_ (c); 100 µmol/L CuCl_2_ (d) and 300 µmol/L CuCl_2_ (e). Comparison of efficacies of 20 µmol/L 3-mercapto-2-methylpentan-1-ol on OR2M3 with different Cu^2+^ concentrations in ascending order (f). Note that the same data set in the absence of supplemented Cu^2+^ (grey) is given in sub-panels (a-e) for didactic reasons. Data were mock control-subtracted, normalized to the OR2M3 wt signal in response to 3-mercapto-2-methylpentan-1-ol (20 µmol/L), measured in the absence of Cu^2+^, and shown as mean ± SD (n = 3 – 6). RLU = relative luminescence unit. 3MMP = 3-mercapto-2-methylpentan-1-ol. Curves represent best fits to the data in the absence (grey) or presence (black) of Cu^2+^, with EC_50_ values given in Table S7. Note that for didactical reasons, the concentration-response relations in Fig. 1d and Fig. S6a, as well as sub-panels Fig. 1c and Fig. S6f, are the identical data set.


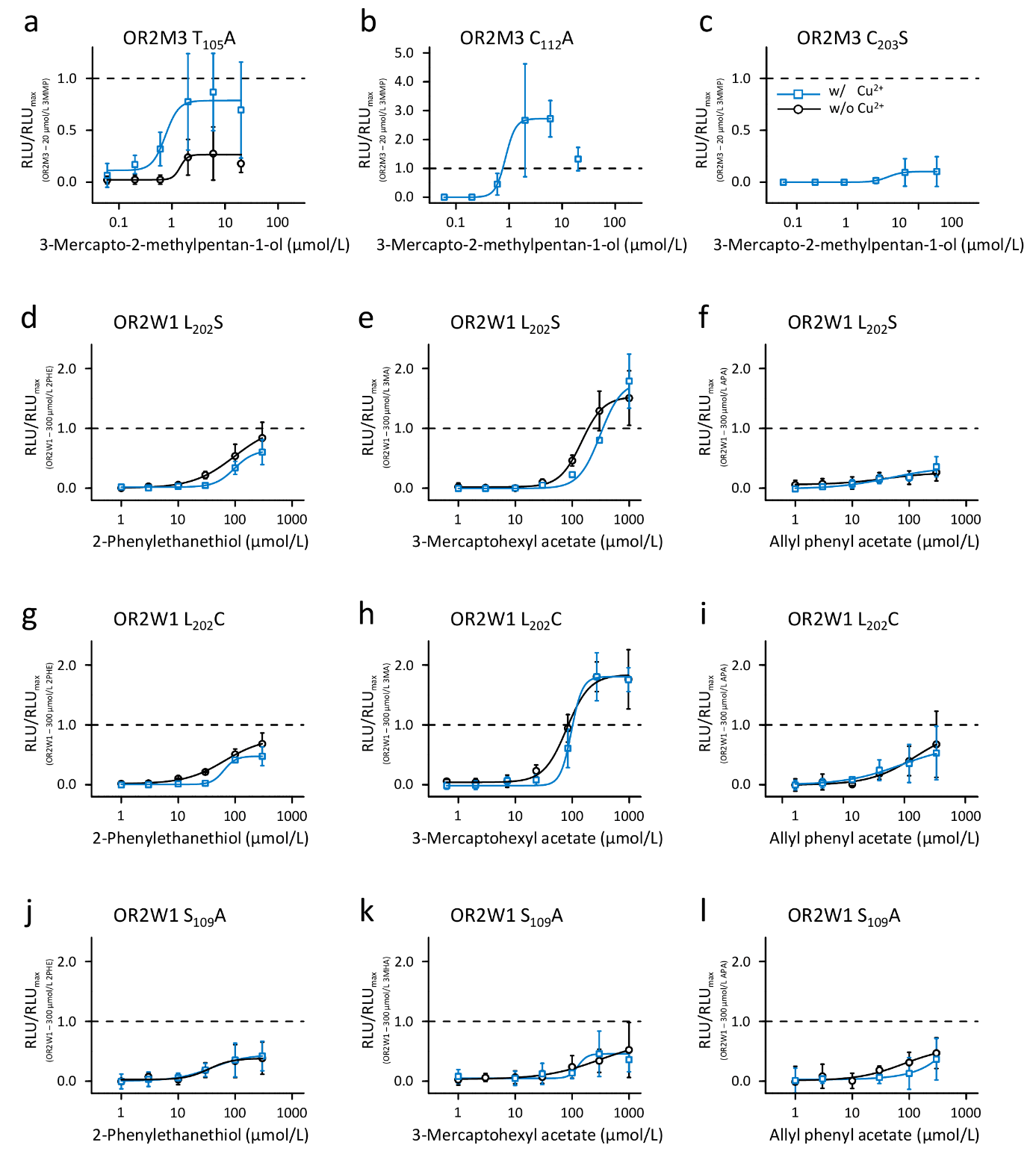


Fig. S8. Cu^2+^ influences the efficacy of 3-mercapto-2-methylpentan-1-ol on OR2M3 but not of OR2W1 agonists.

Effects of 10 µmol/L Cu^2+^ on the concentration-response relations of 3-mercapto-2-methylpentan-1-ol of OR2M3 variants (a, b, c), 2-phenylethanethiol of OR2W1 variants (d, g, j), 3-mercaptohexyl acetate of OR2W1 variants (e, h, k), and allyl phenyl acetate of OR2W1 variants (f, i, l). Data were mock control-subtracted, normalized to each receptor maximum amplitude as response to the respective substance measured in the absence of Cu^2+^, and shown as mean ± SD (n = 3 – 6). RLU = relative luminescence unit. Curves represent best fits to the data in the absence (black) or presence (blue) of Cu^2+^, with EC_50_ values given in Table 4. 3MMP = 3-mercapto-2-methylpentan-1-ol, 2PHE = 2-phenylethanethiol, 3MHAc = 3-mercaptohexyl acetate, APA = allyl phenyl acetate.


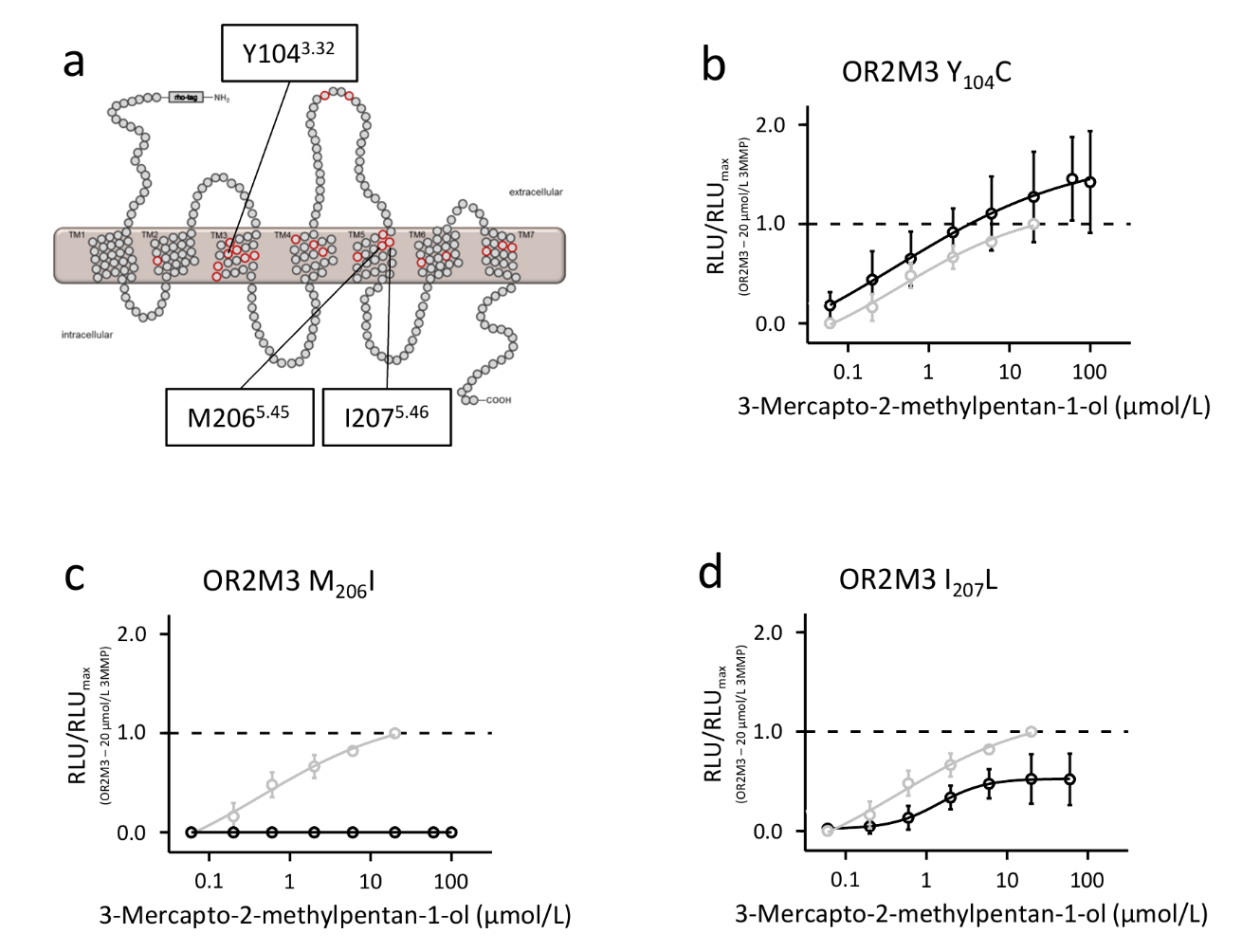


Fig. S9. SNP-defined OR2M3 haplotypes display 3-mercapto-2-methylpentan-1-ol gain- and loss-of-function phenotypes.

(a) Schematic snake diagram with localization of mutated amino acid positions within OR2M3, which have been chosen because of their close vicinity to putative interaction sites with odorants as proposed by Man et al. [80] (red circles). Effects of 3-mercapto-2-methylpentan-1-ol on OR2M3 variants (black curves) with SNP-defined single amino acid changes at positions 104^3.32^ (b), 206^5.45^ (c), and 207^5.46^ (d). Note that the same data set for 3-mercapto-2-methylpentan-1-ol on OR2M3 wt (grey, see also Fig. 1b, d) is given in panels (b)-(d) for didactic reasons. EC_50_ values are given in Table 3. Data were mock control-subtracted, normalized to OR2M3 wt maximum amplitude, and displayed as mean ± SD (n = 3 – 6). RLU = relative luminescence unit. 3MMP = 3-mercapto-2-methylpentan-1-ol.


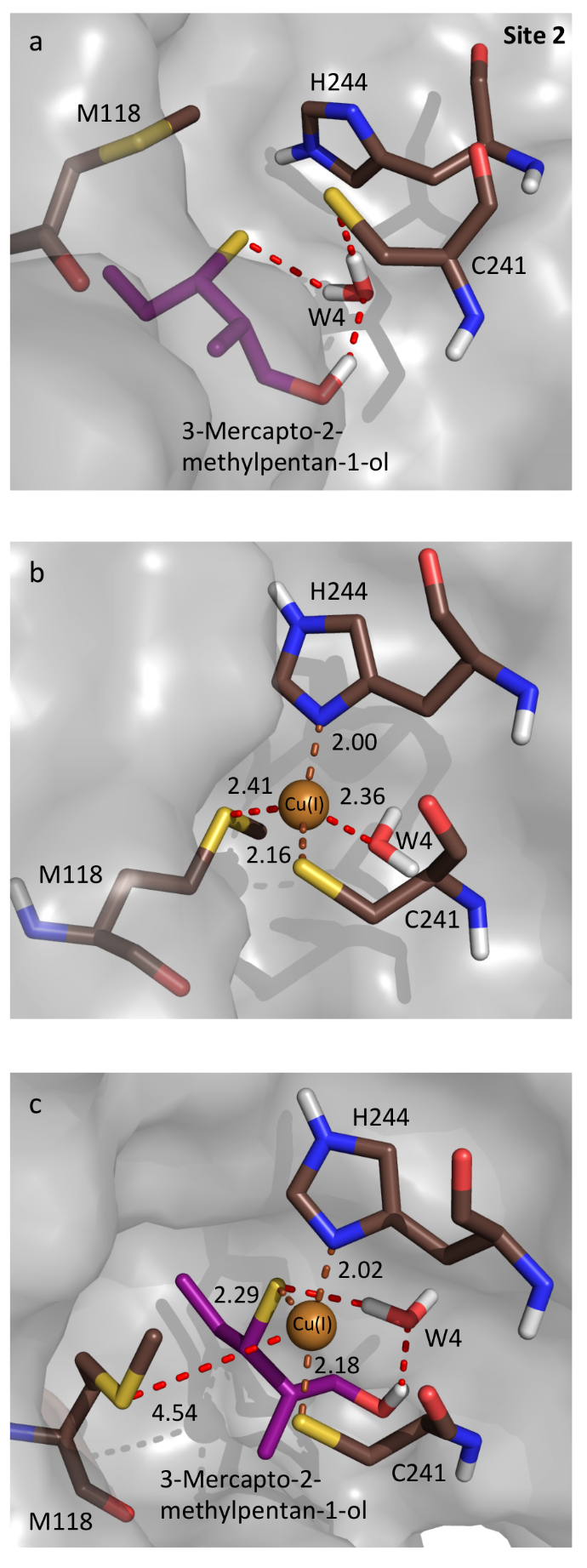


Fig S10. Molecular models of the ligand and copper binding cavity for OR2M3.

QM/MM structural model of site 2 in OR2M3 with the ligand 3-mercapto-2-methylpentan-1-ol (a), with Cu(I) (b), or both (c). Residues defining the binding pocket are shown as sticks (oxygen: red; nitrogen: blue; sulfur: yellow).


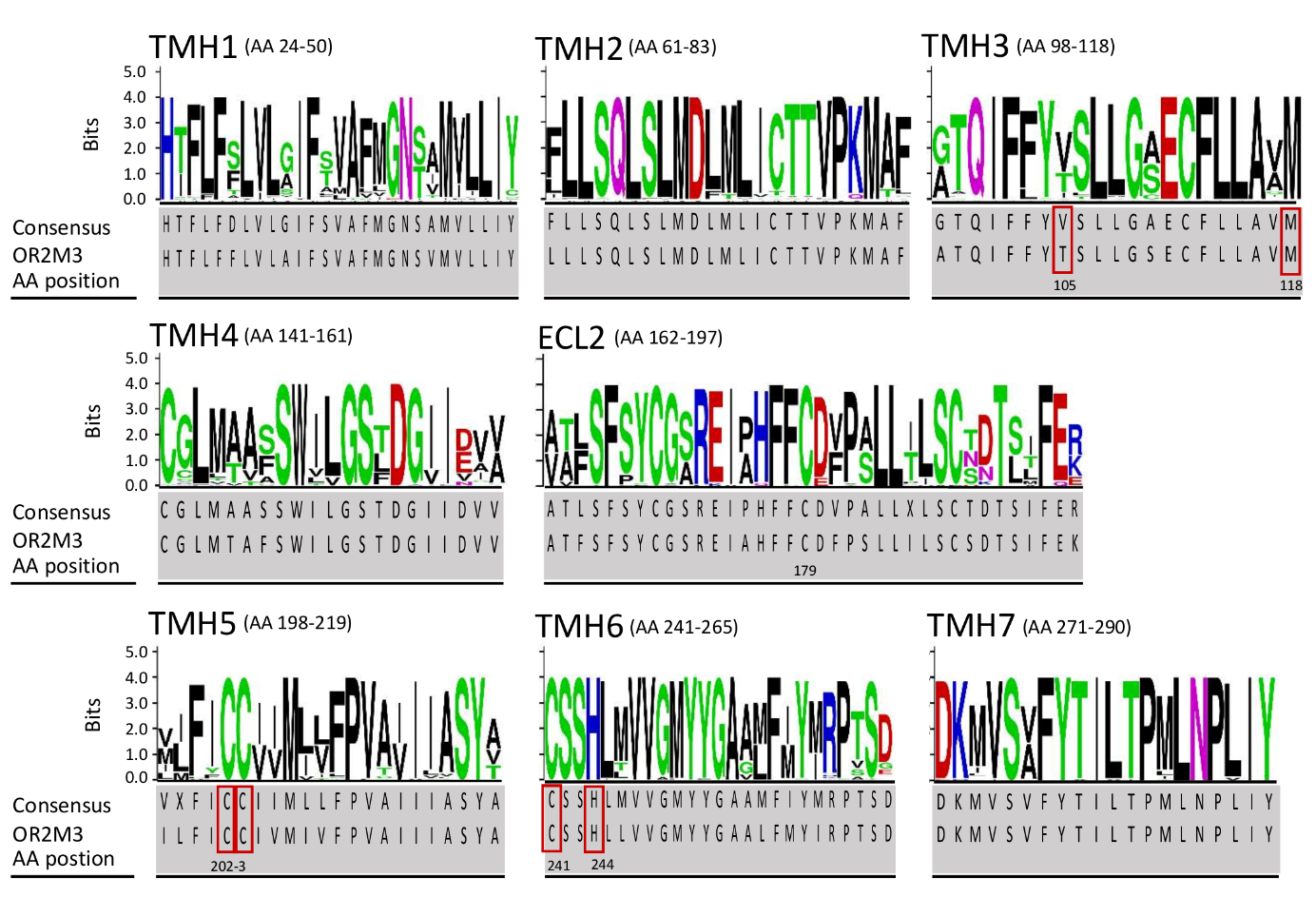


Fig. S11. A conserved copper binding motif within OR2M3 homologs.

Alignments of transmembrane helices (TMH 1 – 7) and extracellular loop 2 (ECL 2) of human OR2M3 and its 47 orthologs and paralogs. Shown are sequence logos, the consensus sequence, and the human OR2M3 sequence with the 3-mercapto-2-methylpentan-1-ol and copper binding pocket (red boxes). The consensus amino acid refers to the most frequent one, which is determined by letter height and stacking order. The letters of each stack are ordered from the most frequent to the least frequent. Amino acid conservation is measured in bits, and a 100% conservation correlates to 4.32 bits [[4](#_ENREF_4)]. Basic amino acids (K, R, H) are blue, polar (G, S, T, Y C) are green, hydrophilic (Q, N) are purple, acidic (D, E) are red, and hydrophobic (A, V, L, I, P, W, M, F) are black. All reference sequences with their accession numbers are given in Table S5.


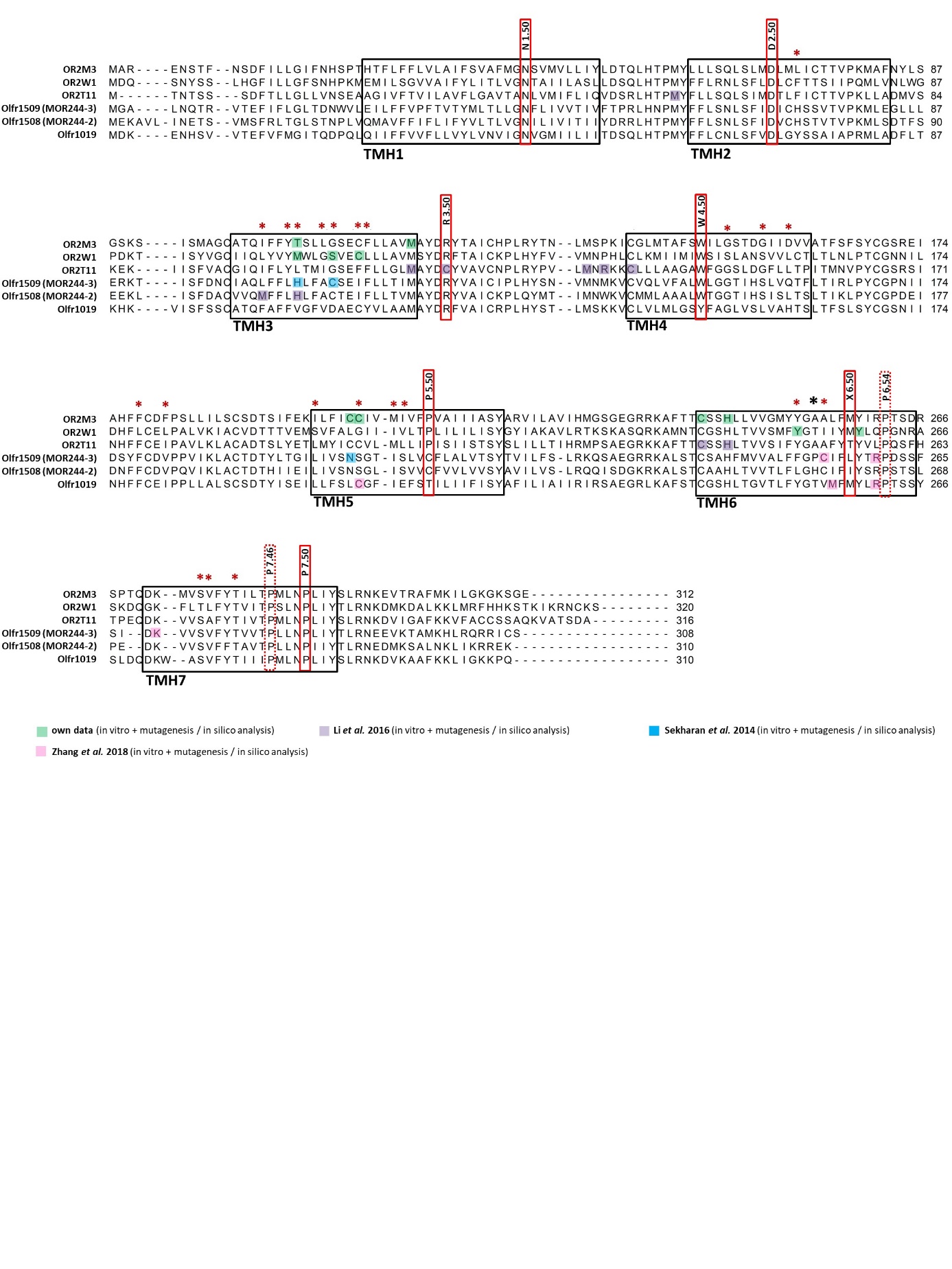


Fig. S12. Copper binding site within different ORs.

Shown is the sequence alignment (CLC Main Workbench 6.5) of ORs which were previously investigated by different working groups [[5-7](#_ENREF_5)]. The respectively identified amino acids of the copper binding site are highlighted with color boxes (see color coding). The transmembrane helices (TMHs) are indicated as black boxes. According to the Ballesteros-Weinstein nomenclature the highly conserved amino acid within each TMH is given the number ‘50’ [[8](#_ENREF_8)]. The 22 amino acid residues according to Man *et al.* [[1](#_ENREF_1)] are marked with red asterisk. The black asterisk in TMH 6 refers to X6.50 according to de March *et al.* [[9](#_ENREF_9), [10](#_ENREF_10)].


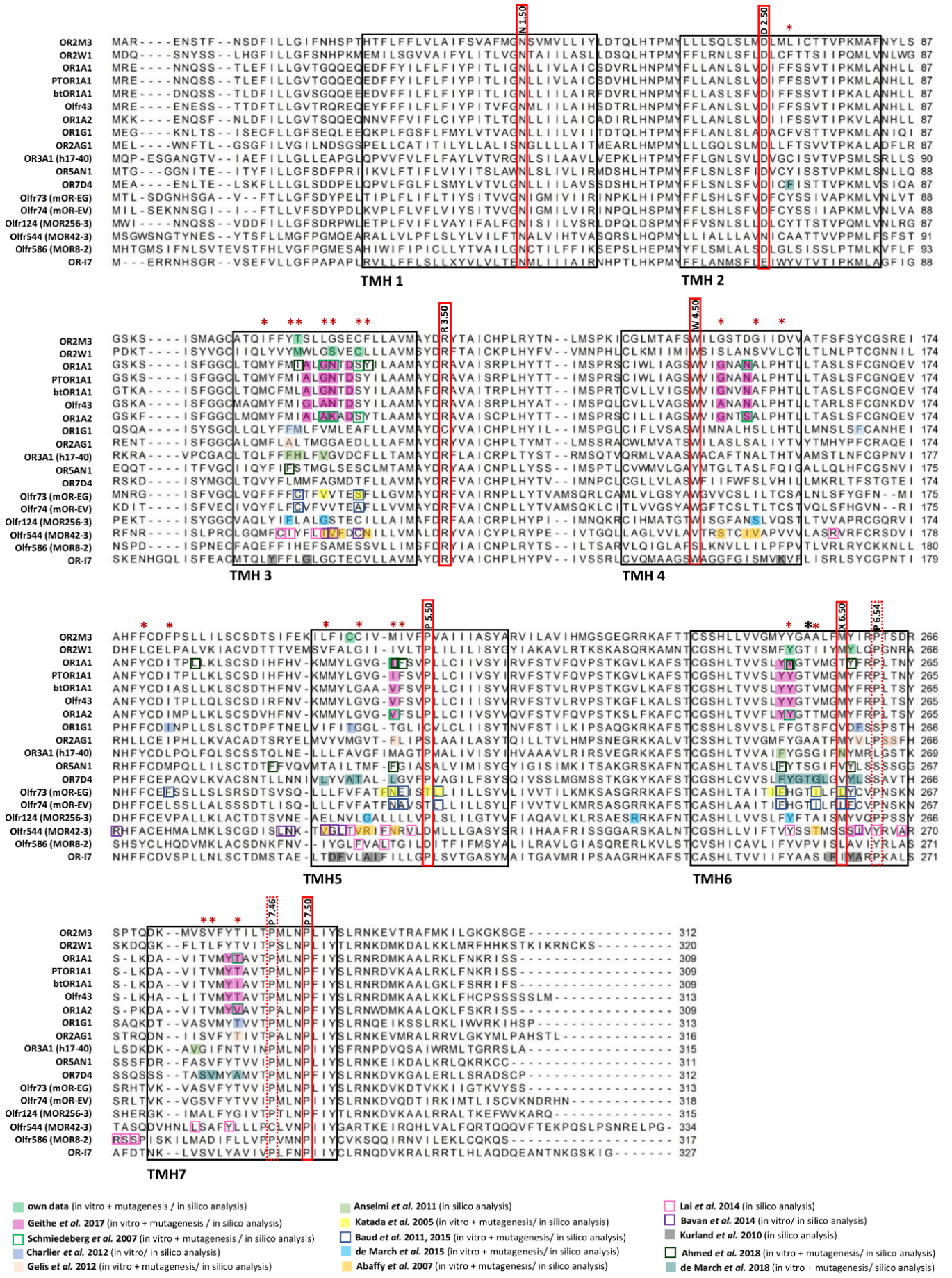


Fig. S13. Odorant binding site within different ORs.

Shown is the sequence alignment (CLC Main Workbench 6.5) of ORs which were previously investigated by different working groups [[10-24](#_ENREF_10)]. The respectively identified amino acids of the odorant binding site were highlighted with color boxes (see color coding). The transmembrane helices (TMHs) are indicated as black boxes. According to the Ballesteros-Weinstein nomenclature the highly conserved amino acid within each TMH was given the number ‘50’ [[8](#_ENREF_8)]. The 22 amino acid residues according to Man *et al.* [[1](#_ENREF_1)] were marked with red asterisk. The black asterisk in TMH 6 refers to X6.50 according to de March *et al.* [[9](#_ENREF_9), [10](#_ENREF_10)].

**Measuring cell surface expression of OR2M3 and its variants by using flow cytometry**

NxG 108CC15 cells [[25](#_ENREF_25)] were cultivated in 12-well plates (80 000 cells/well) and transiently transfected with 800 ng plasmid DNA (pI2-dk – 39AA rho-tag + HaloTag®) of the respective odorant receptor (OR) variant as well as 400 ng plasmid DNA of Gαolf, Gγ13, RTP1S and cAMP-luciferase pGloSensor^TM^-22F each using Lipofectamine® 2000 (Life Technologies, Carlsbad, USA). To keep the amount of transfected DNA compared to cell number the same as in the luminescence assay, we also transfected the cAMP-luciferase pGloSensor^TM^-22F, although it has no impact on cell surface expression of the respective OR. Basically, the experimental settings were scaled up 8-fold from the 96-well luminescence assay to the 12-well flow cytometry assay. The rho-tagged OR were additionally fused with a HaloTag® (Promega, Madison, USA) [[26](#_ENREF_26)] which is suitable for the detection by fluorescence labeled ligands via flow cytometry.

We used the membrane non-permeable ligand HaloTag® AlexaFluor 488 (ex/em = 499/518 nm) and the membrane permeable HaloTag® TMR ligand (ex/em = 552/578 nm) from Promega, Madison, USA.

For analysis, cells were harvested 42 h post transfection and stained for 1 hour either with HaloTag® AlexaFluor 488 ligand or HaloTag® TMR ligand at 37°C, 7% CO_2_ and 100 % humidity. Afterwards, the cells were washed twice with serum free medium prior flow cytometry analysis (MACSQuant Analyzer, Milteny Biotec, Bergisch Gladbach, Germany). For the HaloTag® TMR ligand, an additionaly incubation step in growth medium for 30 min at at 37°C, 7% CO_2_ and 100 % humidity was necessary.

A forward- and side-scatter gate was set to exclude dead cells with forward-scatter (FSC: 235V) and side-scatter (SSC: 360V). The FITC signal (B1-channel; HaloTag® Alexa Fluor488 ligand was detected with 190V and the PE signal (B2-channel, HaloTag® TMR ligand) with 416V. In each case 10,000 cells were measured. The analysis was performed with the MACSQuantify software (Milteny Biotec, Bergisch Gladbach, Germany). The FITC or PE signal of each mock-control defined the distinction between negative and positive cells.

Fig. S14: Cell surface expression of OR2M3 wt and variants.

Shown are mean ± SD (n=3-6). The data represents the percentage of relative membrane expression of OR-transfected NxG cells determined by membrane non-permeable HaloTag® AlexaFluor 488 ligand (dark grey bars) compared to membrane permeable HaloTag® TMR ligand (light grey bars). wt, NCBI reference sequence.

**Supplemental Literature**

1. Man O, Gilad Y, Lancet D (2004) Prediction of the odorant binding site of olfactory receptor proteins by human-mouse comparisons. Protein Sci 13 (1):240-254.

2. Genomes Project C, Abecasis GR, Altshuler D, Auton A, Brooks LD, Durbin RM, Gibbs RA, Hurles ME, McVean GA (2010) A map of human genome variation from population-scale sequencing. Nature 467 (7319):1061-1073.

3. Sherry ST, Ward MH, Kholodov M, Baker J, Phan L, Smigielski EM, Sirotkin K (2001) Dbsnp: The ncbi database of genetic variation. Nucleic Acids Res 29 (1):308-311.

4. Crooks GE, Hon G, Chandonia J-M, Brenner SE (2004) Weblogo: A sequence logo generator. Genome Research 14 (6):1188-1190.

5. Sekharan S, Ertem MZ, Zhuang H, Block E, Matsunami H, Zhang R, Wei JN, Pan Y, Batista VS (2014) Qm/mm model of the mouse olfactory receptor mor244-3 validated by site-directed mutagenesis experiments. Biophys J 107 (5):L5-L8.

6. Li S, Ahmed L, Zhang R, Pan Y, Matsunami H, Burger JL, Block E, Batista VS, Zhuang H (2016) Smelling sulfur: Copper and silver regulate the response of human odorant receptor or2t11 to low-molecular-weight thiols. J Am Chem Soc.

7. Zhang R, Pan Y, Ahmed L, Block E, Zhang Y, Batista VS, Zhuang H (2018) A multispecific investigation of the metal effect in mammalian odorant receptors for sulfur-containing compounds. Chem Senses 43 (5):357-366.

8. Ballesteros JA, Weinstein H (1995) Integrated methods for the construction of three-dimensional models and computational probing of structure-function relations in g protein-coupled receptors. In: Sealfon SC (ed) Methods in neurosciences, vol 25. Academic Press, pp 366-428.

9. de March CA, Kim SK, Antonczak S, Goddard WA, 3rd, Golebiowski J (2015) G protein-coupled odorant receptors: From sequence to structure. Protein Sci 24 (9):1543-1548.

10. de March CA, Yu Y, Ni MJ, Adipietro KA, Matsunami H, Ma M, Golebiowski J (2015) Conserved residues control activation of mammalian g protein-coupled odorant receptors. J Am Chem Soc 137 (26):8611-8616.

11. Geithe C, Protze J, Kreuchwig F, Krause G, Krautwurst D (2017) Structural determinants of a conserved enantiomer-selective carvone binding pocket in the human odorant receptor or1a1. Cell Mol Life Sci 74 (22):4209-4229.

12. Schmiedeberg K, Shirokova E, Weber HP, Schilling B, Meyerhof W, Krautwurst D (2007) Structural determinants of odorant recognition by the human olfactory receptors or1a1 and or1a2. J Struct Biol 159 (3):400-412.

13. Gelis L, Wolf S, Hatt H, Neuhaus EM, Gerwert K (2012) Prediction of a ligand-binding niche within a human olfactory receptor by combining site-directed mutagenesis with dynamic homology modeling. Angew Chem Int Ed Engl 51 (5):1274-1278.

14. Katada S, Hirokawa T, Oka Y, Suwa M, Touhara K (2005) Structural basis for a broad but selective ligand spectrum of a mouse olfactory receptor: Mapping the odorant-binding site. J Neurosci 25 (7):1806-1815.

15. Baud O, Etter S, Spreafico M, Bordoli L, Schwede T, Vogel H, Pick H (2011) The mouse eugenol odorant receptor: Structural and functional plasticity of a broadly tuned odorant binding pocket. Biochemistry 50 (5):843-853.

16. Abaffy T, Malhotra A, Luetje CW (2007) The molecular basis for ligand specificity in a mouse olfactory receptor: A network of functionally important residues. J Biol Chem 282 (2):1216-1224.

17. Lai PC, Crasto CJ (2012) Beyond modeling: All-atom olfactory receptor model simulations. Front Genet 3:61.

18. Anselmi C, Buonocore A, Centini M, Facino RM, Hatt H (2011) The human olfactory receptor 17-40: Requisites for fitting into the binding pocket. Comput Biol Chem 35 (3):159-168.

19. Bavan S, Sherman B, Luetje CW, Abaffy T (2014) Discovery of novel ligands for mouse olfactory receptor mor42-3 using an in silico screening approach and in vitro validation. PLoS One 9 (3):e92064.

20. Kurland MD, Newcomer MB, Peterlin Z, Ryan K, Firestein S, Batista VS (2010) Discrimination of saturated aldehydes by the rat i7 olfactory receptor. Biochemistry 49 (30):6302-6304.

21. Baud O, Yuan S, Veya L, Filipek S, Vogel H, Pick H (2015) Exchanging ligand-binding specificity between a pair of mouse olfactory receptor paralogs reveals odorant recognition principles. Scientific Reports 5:14948.

22. Charlier L, Topin J, Ronin C, Kim SK, Goddard WA, 3rd, Efremov R, Golebiowski J (2012) How broadly tuned olfactory receptors equally recognize their agonists. Human or1g1 as a test case. Cell Mol Life Sci 69 (24):4205-4213.

23. Ahmed L, Zhang Y, Block E, Buehl M, Corr MJ, Cormanich RA, Gundala S, Matsunami H, O'Hagan D, Ozbil M, Pan Y, Sekharan S, Ten N, Wang M, Yang M, Zhang Q, Zhang R, Batista VS, Zhuang H (2018) Molecular mechanism of activation of human musk receptors or5an1 and or1a1 by (*r*)-muscone and diverse other musk-smelling compounds. Proc Natl Acad Sci U S A 115 (17):E3950-E3958.

24. de March CA, Topin J, Bruguera E, Novikov G, Ikegami K, Matsunami H, Golebiowski J (2018) Odorant receptor 7d4 activation dynamics. Angew Chem Int Ed Engl 57 (17):4554-4558.

25. Hamprecht B, Glaser T, Reiser G, Bayer E, Propst F (1985) Culture and characteristics of hormone-responsive neuroblastoma x glioma hybrid cells. Methods Enzymol 109:316-341.

26. Los GV, Encell LP, McDougall MG, Hartzell DD, Karassina N, Zimprich C, Wood MG, Learish R, Ohana RF, Urh M, Simpson D, Mendez J, Zimmerman K, Otto P, Vidugiris G, Zhu J, Darzins A, Klaubert DH, Bulleit RF, Wood KV (2008) Halotag: A novel protein labeling technology for cell imaging and protein analysis. ACS Chem Biol 3 (6):373-382.
